# Selective behavioral and prefrontal transcriptomic correlates of acute clozapine exposure in socially isolated mice

**DOI:** 10.3389/fphar.2026.1944363

**Published:** 2026-09-28

**Authors:** Youzhi Xu, Ao Gao, Yixuan Zhang, Zishen Zhang, Yanzhi Zhao, Chunyue Huo

**Affiliations:** 1 Capital Medical University Yanjing Medical College, Beijing, China; 2 Ciqu Community Healthcare Center of Tongzhou District, Beijing, China

**Keywords:** acute pharmacological challenge, clozapine, prefrontal cortex, RNA sequencing, social isolation rearing

## Abstract

**Background:**

Clozapine is an effective antipsychotic for treatment-resistant schizophrenia, but acute molecular responses to clozapine remain incompletely characterized. Social isolation rearing produces schizophrenia-relevant behavioral abnormalities and permits joint evaluation of housing and drug exposure.

**Objective:**

To characterize behavioral and prefrontal transcriptomic correlates of an acute clozapine challenge in socially isolated mice, reassess previously prioritized *Ank3* and *Nrxn1*, and provide external cellular-context hypotheses for candidate genes.

**Methods:**

Male C57BL/6J mice were evaluated in a four-group housing-by-drug design using open-field, novel-object recognition and prepulse-inhibition tests. For a reproducible transcriptome-wide audit, 11 publicly retrievable prefrontal cortex libraries were re-quantified using a factorial DESeq2 model with false-discovery-rate control. Historical pairwise prioritization, network analysis, machine-learning screening, molecular docking and molecular-dynamics simulations were treated as exploratory analyses. Adult wild-type prefrontal cortex single-cell RNA-seq data were used for cellular localization, CellChat analysis and virtual knockout, whereas an embryonic cortical reference was used separately for CellOracle transcription-factor perturbation.

**Results:**

Acute clozapine showed its clearest behavioral effect on isolation-associated hyperlocomotion, whereas recognition memory was not improved and prepulse-inhibition results varied by stimulus intensity. The primary DESeq2 analysis identified 56 housing-by-drug interaction genes at FDR <0.05; *Lamb2* was the strongest FDR-supported transcriptomic signal. *Nrxn1* showed directionally opposite changes in historical pairwise contrasts but did not reach FDR significance in the factorial model, whereas *Ank3* showed no corresponding factorial support. In adult prefrontal cortex single-cell data, *Lamb2* was concentrated in endothelial cells, while *Ank3* and *Nrxn1* were broadly detected in neuronal populations. Virtual *Ank3* and *Nrxn1* knockout profiles contained 16 and 15 significant non-target genes, respectively, with five shared genes and no significant reciprocal candidate effect. Embryonic CellOracle analyses identified model-dependent upstream transcription-factor sensitivity of *Ank3* and *Nrxn1*.

**Conclusion:**

These findings define selective behavioral and prefrontal transcriptomic correlates of an acute clozapine challenge rather than generalized behavioral rescue or clinical treatment-response prediction. *Lamb2* was the strongest FDR-supported signal in the primary model, whereas *Ank3* and *Nrxn1* remain exploratory computational candidates. The external single-cell and virtual-perturbation analyses provide testable cellular and regulatory hypotheses, not evidence of direct binding, causal regulation or clozapine-induced effects *in vivo*.

## 1 Introduction

Schizophrenia is a severe psychiatric disorder characterized by positive, negative and cognitive symptoms. Clozapine remains the most effective antipsychotic for many people with treatment-resistant schizophrenia (Schneider-Thoma et al., 2025), yet its clinically relevant actions emerge during sustained treatment and cannot be reduced to the transcriptional response after a single dose. Acute experimental paradigms are nevertheless useful for separating immediate drug-associated effects from housing-related molecular differences, provided that they are not described as models of clinical response prediction.

Mechanistic studies of clozapine response face a central design problem: molecular differences after drug exposure do not by themselves distinguish therapeutic response from acute pharmacology, disease state or nonspecific motor effects. Social isolation rearing produces reproducible behavioral abnormalities relevant to schizophrenia (Jones et al., 2011; Zhang et al., 2022), and the prefrontal cortex is a relevant tissue for studying schizophrenia-associated behavioral and transcriptional phenotypes (Zhang et al., 2021; Huo et al., 2018). This model can therefore identify acute housing-dependent drug correlates, but it cannot establish clinical response biomarkers without responder heterogeneity and independent validation.

An acute challenge design provides a controlled approach for examining early behavioral and prefrontal molecular correlates of clozapine exposure. Acute clozapine administration can induce early molecular changes in rodent brain tissue (Robbins et al., 2008; Korlatowicz et al., 2021), but the relationship between these short-term effects and prefrontal transcriptional changes remains incompletely defined. Integrating a factorial behavioral design with transcriptome-wide reanalysis and exploratory computational prioritization can distinguish robust housing-by-drug interaction signals from candidate patterns requiring further validation. The aim of the present study was not to identify a clinical response biomarker, but to define experimentally testable molecular correlates and generate hypotheses for subsequent mechanistic studies.

Here, we combined behavioral testing with PFC RNA sequencing to examine immediate correlates of acute clozapine exposure in socially isolated mice. The primary questions were whether the behavioral effect depended on housing condition and which transcriptomic signals showed a housing-by-drug interaction or an opposite-direction change after clozapine. Network analysis, feature selection, structural simulation, external single-cell reannotation, cell-communication analysis and virtual perturbation were treated as exploratory hypothesis-generating layers rather than independent validation.

## 2 Materials and methods

### 2.1 Study approval

Animal care and treatment were approved by the Capital Medical University Institutional Animal Care and Use Committee (IACUC number: AEEI-2020–166) and followed “Guide for the Care and Use of Laboratory Animals”.

### 2.2 Animal

C57BL/6J male mice were supplied by Beijing Vital River Laboratory Animal Technology Co., Ltd (Beijing, China). At postnatal day (PND) 21, 38 healthy male mice were housed in the SPF animal facility at Yanjing Medical College, Capital Medical University. Mice were maintained at 24 °C ± 2 °C and 55% ± 15% humidity under a 12-h light/dark cycle, with lights on at 8:00 a.m. Food and water were available *ad libitum*. Behavioral procedures were conducted from P49 to P56 between 8:00 a.m. and 5:00 p.m. Efforts were made to minimize animal number and suffering.

The study was designed as a male-only implementation of an established social-isolation rearing paradigm for schizophrenia-relevant behavioral phenotypes (Jones et al., 2011; Zhang et al., 2022). Restricting the exploratory cohort to one sex maintained consistency with the established housing, behavioral and tissue-collection procedures. This design does not permit assessment of sex-dependent effects; therefore, the findings are restricted to male mice.

### 2.3 Animal model construction

Immediately after weaning at P21, mice were assigned to two housing conditions: a social-reared control condition (SOC; four mice per cage, n = 20) or social isolation rearing (SIR; one mouse per cage, n = 18). Individually housed mice retained visual, auditory, and olfactory contact with neighboring mice but had no direct physical contact. Both housing groups were maintained in the same controlled room, and social isolation continued for 4 weeks.

Behavioral assessment was conducted from P49 to P56. During the subsequent drug-phase assessment, each housing condition included vehicle- and clozapine-treated animals, yielding four behavioral groups: SOC-vehicle (n = 10), SOC-clozapine (n = 10), SIR-vehicle (n = 9), and SIR-clozapine (n = 9). In the original archived analysis files, the label “IRZ” referred to the SIR-clozapine group. Thus, IRZ was not an additional experimental group but an alternative label for SIR-clozapine. The revised inferential analysis used the complete four-group housing-by-drug design.

The behavioral battery was performed in the order open-field test (OFT), novel object recognition test (NOR), and prepulse inhibition (PPI), with PPI conducted last because it was the most stressful procedure. On P56, immediately after completion of the final behavioral session, mice were euthanized and prefrontal cortex tissue was collected for molecular analysis. The overall experimental and analytical workflow is summarized in Figure 1, and the experimental timeline is shown in Figure 2A.

**FIGURE 1:**
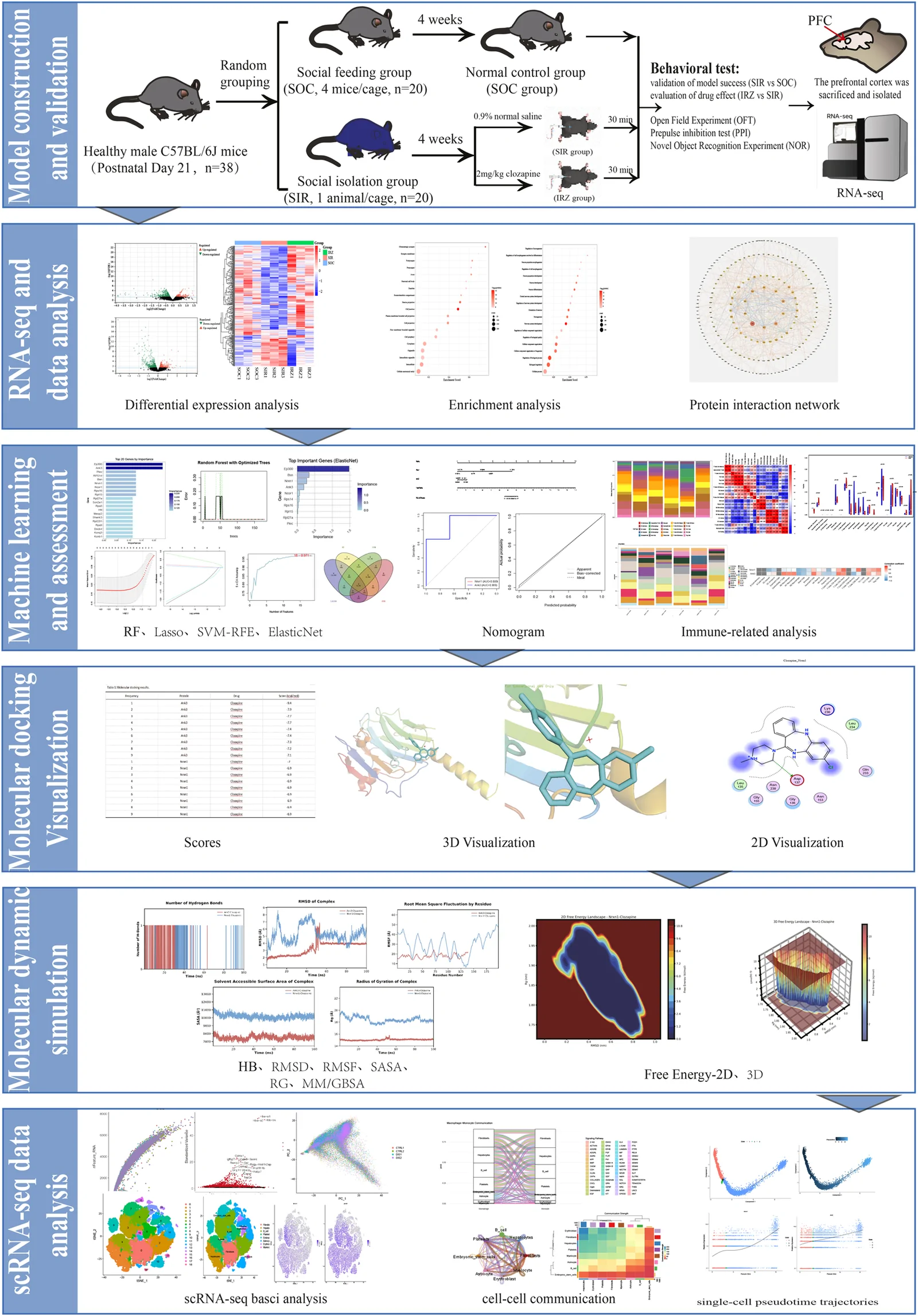
The overall workflow of this study.

**FIGURE 2:**
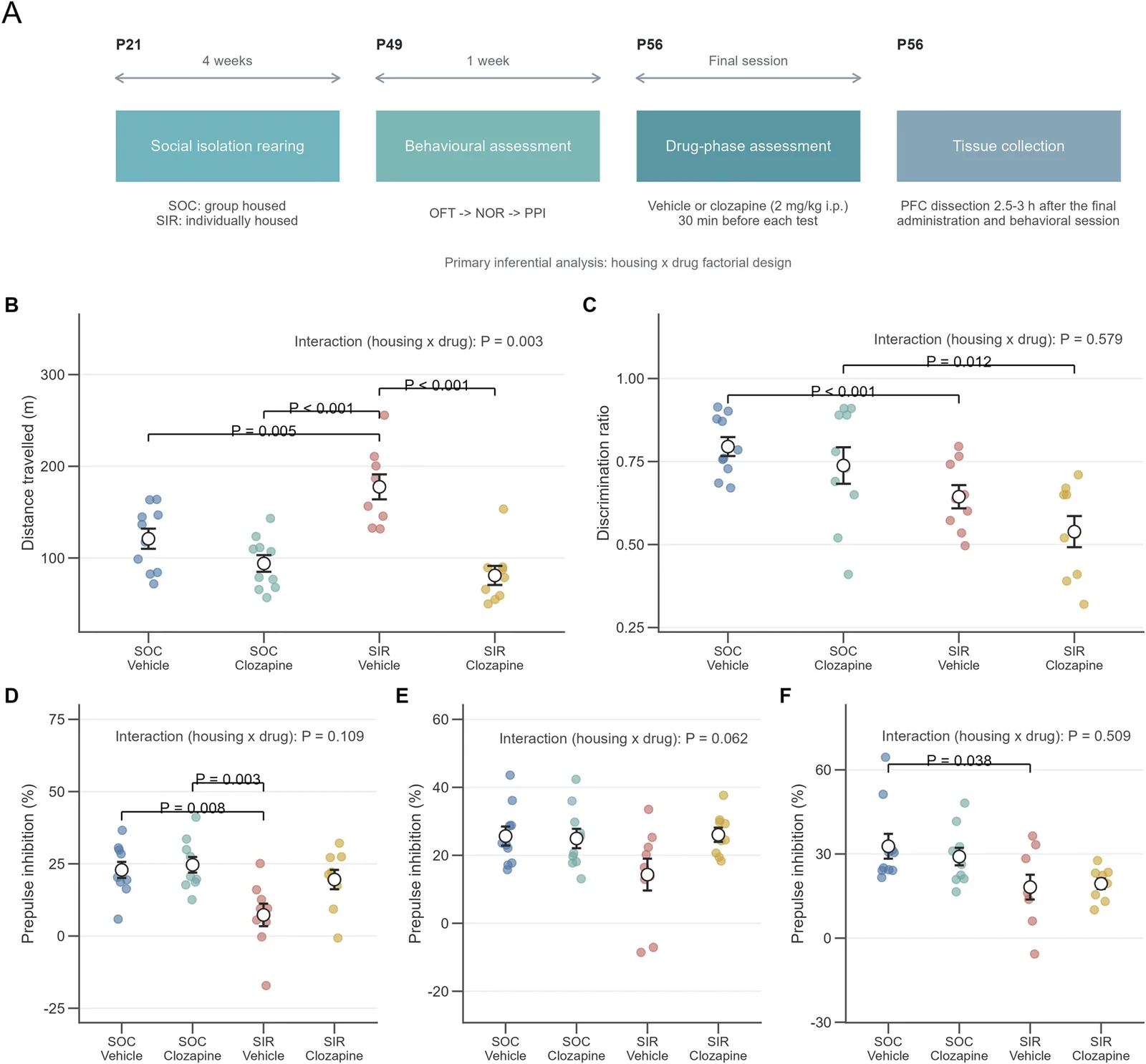
Factorial analysis of behavioral effects associated with acute clozapine exposure **(A)** Experimental timeline. **(B)** Open-field distance travelled. **(C)** Novel-object recognition discrimination ratio. **(D–F)** Prepulse inhibition at 69, 78 and 88 dB, respectively. Each point represents one mouse; open circles and error bars indicate mean ± SEM. All outcomes were analyzed using two-way ANOVA with housing and drug as between-subject factors. Interaction P values are displayed in each panel. Brackets indicate Tukey-adjusted pairwise comparisons shown in the panel. SOC, socially group-housed; SIR, socially isolated rearing.

### 2.4 Drug treatments and animal grouping

Clozapine (Sigma-Aldrich, United States) was prepared in sterile 0.9% saline. During the drug-phase behavioral assessment, mice received clozapine (2 mg/kg, i. p.) or an equivalent volume of vehicle (0.9% saline, 2 mL/kg) 30 min before each behavioral test. Thus, each administration represented an acute pretreatment for the corresponding test; the protocol was not designed to model sustained clinical clozapine therapy. For clarity, the original candidate-gene workflow and the revised factorial analysis used different comparison frameworks. The original workflow generated candidate genes from two prespecified pairwise contrasts: SOC versus SIR under vehicle conditions, representing the housing-associated contrast, and SIR-vehicle versus SIR-clozapine, representing the clozapine-associated contrast within socially isolated mice. The SOC-clozapine group was present in the experiment but was not included in this historical pairwise-overlap criterion. In the revised analysis, all four groups were included in a factorial model to estimate the main effects of housing, drug treatment, and their interaction.

### 2.5 Behavioral testing

Behavioral tests were performed in the following order: open-field test (OFT), novel object recognition (NOR) test, and prepulse inhibition (PPI) test. PPI was conducted last because it is relatively stressful.

#### 2.5.1 Open field test

The OFT was used to assess locomotor activity (Huo et al., 2018). Mice were transferred to a quiet, dimly lit testing room 1 h before testing. Each mouse was placed at the edge of a 50 × 50 cm open-field chamber and was allowed to explore for 30 min. Behavior was recorded using an infrared activity-monitoring system with Xeye Aba software (Beijing, China). Total distance travelled during 30 min was used to quantify locomotor activity.

#### 2.5.2 Novel object recognition test

The NOR task was used to evaluate recognition memory. Mice were transferred to a quiet, dimly lit room 1 h before testing. The task consisted of habituation, familiarization, and test sessions. During habituation, each mouse explored the empty arena for 5 min. During familiarization, two identical objects were presented for 5 min. After a 1-h interval, one familiar object was replaced with a novel object, and exploration was recorded for 5 min. Object position was counterbalanced, and an observer blinded to housing and treatment scored exploration time. The apparatus and objects were cleaned with 75% ethanol and dried between trials.

The discrimination ratio was calculated as TN/(TN + TF), where TN is the time spent exploring the novel object and TF is the time spent exploring the familiar object.

#### 2.5.3 Prepulse inhibition

PPI was assessed using the Xeye Startle Reflex system (Beijing, China). Mice were acclimated to the testing room for 1 h. A 65-dB background noise was maintained throughout the session. Pulse-alone trials consisted of a 110-dB, 40-m acoustic stimulus. Prepulse-plus-pulse trials consisted of a 20-m prepulse at 69, 78, or 88 dB followed by the 110-dB pulse. Pulse-alone, prepulse-alone, prepulse-plus-pulse, and no-stimulus trials were presented in pseudorandom order, with each trial type repeated eight times and intertrial intervals varying from 8 to 20 s.

PPI was calculated as 100 × [1 - (startle amplitude during prepulse-plus-pulse trials/startle amplitude during pulse-alone trials)]. The apparatus was cleaned with 75% ethanol and dried between animals.

### 2.6 Prefrontal tissue collection

At the end of the final behavioral session on P56, mice were deeply anesthetized by intraperitoneal injection of sodium pentobarbital (50 mg/kg body weight). The depth of anesthesia was confirmed by the absence of a pedal reflex. Euthanasia was then performed by cervical dislocation, and death was confirmed before tissue collection. According to the experimental records, PFC tissue was collected approximately 2.5–3 h after the final acute clozapine or vehicle administration. The tissue was rapidly dissected, placed on dry ice and stored at −80 °C until processing. This sampling schedule captured a post-behavioral acute-exposure state rather than a steady-state treatment effect or a molecular signature of long-term clinical response.

Because the RNA-seq component was limited to a subset of the behavioral cohort, mice within each experimental group were assigned numeric identifiers and three animals per group were selected using a random-number table. Thus, RNA-seq samples were not selected on the basis of behavioral performance. The retained records identify the sequenced library labels as SOC1–3PFC, SIR1–3PFC and IRZ1–3PFC, with IRZ referring to the SIR-clozapine group. For the reproducible complete-matrix audit, 11 publicly retrievable PFC libraries were reprocessed, comprising SOC-vehicle (n = 3), SIR-vehicle (n = 3), SOC-clozapine (n = 3), and SIR-clozapine (n = 2). The locally documented but publicly unavailable IRZ3PFC library was not imputed.

### 2.7 RNA-seq

For clarity, this experiment used bulk RNA sequencing rather than single-cell RNA sequencing; therefore, a total cell count was not generated. The reproducible complete-matrix audit included 11 publicly retrievable prefrontal cortex libraries: SOC-vehicle (n = 3), SOC-clozapine (n = 3), SIR-vehicle (n = 3), and SIR-clozapine (n = 2). Total RNA was extracted from prefrontal cortex tissue using the E. Z.N.A. Total RNA Kit I (Omega Bio-tek, Norcross, GA, United States). mRNA libraries were prepared using the VAHTS mRNA-seq V2 Library Prep Kit for Illumina (Vazyme Biotech Co., Ltd., Nanjing, China) and sequenced as paired-end reads on an Illumina NovaSeq 6,000 platform. Adapter-containing and low-quality reads were removed using Trimmomatic v0.39 (Bolger et al., 2014). Reads were aligned to the *Mus musculus* GRCm39, Ensembl release 113 reference genome using HISAT2 v2.1.0 (Kim et al., 2015), and StringTie v2.1.4 was used for transcript assembly and gene-structure optimization (Pertea et al., 2015). The original local pairwise count/FPKM exports retained three biological replicates for each historically analyzed group but contained only nominal-P-filtered genes. They were therefore used to document sample provenance and the original candidate-prioritization workflow, not to reconstruct transcriptome-wide FDR statistics or principal-component analysis. Raw reads associated with BioProject PRJNA1014397 were independently reprocessed for the complete-matrix audit described in Section 2.9.1.

### 2.8 External single-cell RNA-seq datasets

Two external mouse prefrontal cortex single-cell RNA-seq resources were used for complementary computational analyses. Adult wild-type PFC data from GSE181021 were used for major cell-type annotation, candidate-gene localization, neuronal subclustering, CellChat analysis and adult neuronal virtual knockout. The two wild-type samples used in the present analysis contained 5,303 cells after matching the archived cell annotations and expression matrices. This dataset was not exposed to clozapine and was therefore used to provide cellular-context information rather than treatment-response validation.

GSE274763 was analyzed separately as an embryonic cortical reference. The four E15.5 samples included control and As3mtd2d3-transgenic cortical tissues. This dataset was not exposed to clozapine and was not used for adult PFC cell localization or CellChat analysis. It was retained only for motif-constrained upstream transcription-factor perturbation using CellOracle.

### 2.9 Data analysis for gene expression

#### 2.9.1 RNA-seq data analysis process

The 11 publicly archived PFC paired-end libraries were quantified uniformly with kallisto v0.50.1 (Bray et al., 2016) against the *Mus musculus* GRCm39, Ensembl release 113. Transcript abundance was aggregated to the gene level using tximport 1.40.0 with length-scaled counts (Soneson et al., 2015). Genes with at least 10 counts in two or more samples were retained, yielding 16,006 genes entering the DESeq2 analysis. Differential expression was modeled with DESeq2 1.52.0 (Love et al., 2014) using the factorial design housing + drug + housing-by-drug interaction. The primary contrasts estimated the SIR effect under vehicle, the clozapine effect within SOC, the clozapine effect within SIR, and the interaction term. Variance-stabilized counts and the 500 most variable genes were used for principal-component analysis. All samples were retained in the primary model; prespecified sensitivity analyses omitted the PCA-atypical SIR2PFC and SOC3PFC samples separately and jointly, and leave-one-out analyses assessed candidate-gene influence. As sensitivity analyses, the same factorial design was also fitted using the edgeR quasi-likelihood pipeline (Chen et al., 2016) and limma-voom (Law et al., 2014). These analyses were used to assess the robustness of the DESeq2 results under alternative dispersion and variance-modeling assumptions. A complete list of software, versions, sources, and analytical uses is provided in Supplementary Table S3.

To assess the functional coherence of the primary factorial signal, over-representation analyses were performed using clusterProfiler (Yu et al., 2012) for the 56 genes showing an FDR-significant housing-by-drug interaction. Gene Ontology (GO) biological process, cellular component and molecular function terms, as well as Kyoto Encyclopedia of Genes and Genomes (KEGG) pathways, were tested separately. The 16,006 genes retained in the primary DESeq2 analysis were used as the background universe, and P values were adjusted using the Benjamini–Hochberg procedure.

#### 2.9.2 Historical nominal-P pairwise candidate-gene workflow

The original candidate-gene workflow was reconstructed from the archived pairwise analysis files. It used two nominal-P-based comparisons: SOC-vehicle versus SIR-vehicle, representing the housing-associated contrast, and SIR-vehicle versus SIR-clozapine, representing the clozapine-associated contrast within socially isolated mice. In the archived files, the latter comparison was labelled “SIR versus IRZ”, where IRZ referred to the SIR-clozapine group. Genes meeting the original nominal significance criterion in both comparisons were retained, yielding an exploratory overlap of 286 genes. This historical overlap was used for downstream enrichment, protein-interaction analysis and computational prioritization. It was not generated from the revised four-group factorial interaction model and was not treated as an FDR-controlled discovery set.

Gene Ontology and Kyoto Encyclopedia of Genes and Genomes enrichment analyses were performed for the 286-gene exploratory set using clusterProfiler (Yu et al., 2012). The tested-gene universe was defined by the genes retained after expression filtering in the reprocessed RNA-seq dataset, and enrichment P values were adjusted using the Benjamini–Hochberg procedure. Because the input gene set originated from archived nominal-P-based pairwise results, these enrichment analyses were interpreted as exploratory.

#### 2.9.3 Protein interaction network construction and hub gene screening

The historical nominal-P-filtered SIR-vehicle versus SIR-clozapine candidate list was mapped to STRING (Szklarczyk et al., 2023) and visualized in Cytoscape (Shannon et al., 2003). Network degree and centrality were used only to reduce the candidate space for exploratory follow-up. Network position was not interpreted as evidence of causal drug response.

#### 2.9.4 Machine learning-based screening of key genes

Random forest, support-vector-machine recursive feature elimination, LASSO and ElasticNet were applied to the locally retained six-sample SIR-vehicle versus SIR-clozapine expression matrix (Breiman, 2001; Guyon et al., 2002; Tibshirani, 1996; Zou and Hastie, 2005). Their intersection prioritized *Ank3* and *Nrxn1* for exploratory follow-up. Because the matrix had undergone nominal-P-value filtering before feature selection, and because feature selection and evaluation were performed within the same small matrix, these analyses were treated as candidate prioritization rather than unbiased prediction or transcriptomic validation.

#### 2.9.5 Exploratory evaluation of candidate clozapine-responsive genes

Candidate expression, ROC curves, logistic-regression output and the nomogram were retained only as descriptive within-dataset visualizations. No threshold was interpreted as diagnostic, prognostic or predictive, and no claim of calibration or clinical utility was made without an independent validation cohort.

#### 2.9.6 Exploratory immune-signature scoring

The CIBERSORT algorithm (Newman et al., 2015) was run with a mouse-specific immune marker matrix to generate exploratory signature scores. Owing to the small sample size and cortical tissue context, the output was not interpreted as validated immune-cell fractions. Candidate-gene correlations were treated as descriptive and were excluded from the primary mechanism.

#### 2.9.7 Molecular docking

Clozapine was prepared from its PubChem structure by energy minimization, addition of polar hydrogens, assignment of Gasteiger charges and definition of rotatable bonds. Molecular docking was used as an exploratory structure-based screening approach (Stanzione et al., 2021). The *Ank3*-side analysis used the Ankyrin-G-containing PDB structure 5YIP; the *Nrxn1*-side analysis used the locally archived *Nrxn1* structural model. Receptors were prepared in AutoDockTools 1.5.6 (Morris et al., 2009) and exported in PDBQT format. AutoDock Vina (Trott and Olson, 2010) used exhaustiveness 8. The *Ank3* grid was centered at (−17.8684, 57.5704, 66.7892) Angstrom with dimensions (35.9449, 45.4371, 40.9196) Angstrom; the *Nrxn1* grid was centered at (−6.9325, 36.6682, 64.7666) Angstrom with dimensions (64.9895, 39.1220, 56.5593) Angstrom. Ranked poses were treated as computational hypotheses. The receptor files represent a domain-containing experimental structure or a structural model rather than full-length experimentally resolved *Ank3* and *Nrxn1* proteins.

#### 2.9.8 Molecular dynamics simulation

Amber 24 was used to evaluate the structural stability of the modeled *Ank3*–clozapine and *Nrxn1*–clozapine complexes. The systems were prepared using the LEaP module. The protein components were parameterized with the ff14SB force field, whereas clozapine was parameterized using the General Amber Force Field (GAFF). Each complex was solvated in a TIP3P water box with a minimum distance of 10.0 Å between the solute and the box boundary. Sodium and chloride ions were added to neutralize the systems.

A two-stage energy-minimization protocol was applied. During the first stage, positional restraints were retained on the protein backbone, while solvent molecules and ions were minimized. During the second stage, all restraints were removed and the complete systems were minimized using successive steepest-descent and conjugate-gradient procedures.

The minimized systems were gradually heated to 300 K under constant-volume (NVT) conditions with backbone restraints. The systems were then equilibrated under constant-pressure (NPT) conditions at 300 K and 1 bar. A 100-ns unrestrained production simulation was subsequently performed for each complex under NPT conditions.

The trajectories were analyzed with the CPPTRAJ module of Amber. Root-mean-square deviation (RMSD), root-mean-square fluctuation (RMSF), radius of gyration, solvent-accessible surface area, and hydrogen-bonding profiles were calculated to characterize the conformational behavior of the modeled complexes. Molecular mechanics/generalized Born surface area (MM/GBSA) calculations were performed using 100 frames sampled from the final 1 ns of each trajectory. The resulting energy estimates were treated as computational descriptors of the modeled complexes and were not interpreted as experimental evidence of direct clozapine binding.

#### 2.9.9 Adult PFC single-cell reanalysis and cell-cell communication analysis

Adult wild-type mouse PFC single-cell RNA-seq data from GSE181021 were analyzed using Seurat v5.5.1 (Stuart et al., 2019). Two wild-type samples (GSM5482102 and GSM5482103) comprising 5,303 archived annotated cells were retained. The author-provided major cell annotations were checked against canonical markers, including Slc17a7 for excitatory neurons, Gad1 and Gad2 for inhibitory neurons, Pecam1 and Kdr for endothelial cells, Aldh1l1 for astrocytes, Pdgfra for oligodendrocyte precursor cells, and Mbp for oligodendrocytes. Expression of *Lamb2*, *Ank3* and *Nrxn1* was visualized across major cell classes.

Excitatory and inhibitory neurons were subsequently subsetted for secondary analysis. After log normalization, variable-feature selection, regression of total RNA counts and mitochondrial proportion, principal-component analysis, graph-based clustering and UMAP embedding, neuronal subtypes were annotated using canonical markers including Rorb, Foxp2, Oprk1, Slc17a8, Cxcl12, Wfs1, Sst, Gpr88 and Npy.

Cell-cell communication was inferred with CellChat (Jin et al., 2021) using the mouse ligand-receptor database. Major cell-class analyses were performed on all 5,303 adult PFC cells, and neuronal communication analyses were performed in the neuronal subset. Interactions were filtered using a minimum of 10 cells per group. *Lamb2*-associated LAMININ signaling and *Nrxn1*-associated contact-communication patterns were displayed as computational cellular-context outputs. As GSE181021 was not exposed to clozapine, these analyses were not interpreted as treatment-induced communication changes.

### 2.10 In silico perturbation analysis

Virtual knockout of *Ank3* and *Nrxn1* was performed with scTenifoldKnk v1.0.3 (Osorio et al., 2022) using the adult wild-type PFC neuronal subset from GSE181021, comprising 1,747 excitatory and inhibitory neurons. Genes detected in at least 5% of cells were considered eligible, and the 500 most variable eligible genes were retained. *Ank3* and *Nrxn1* were explicitly included in the final input matrix. Three bootstrap-sampled gene regulatory networks were reconstructed using 300 cells per network, three tensor components and two manifold dimensions, with random seed 20260812. Differentially regulated non-target genes were defined using a Benjamini–Hochberg false discovery rate (FDR) threshold of 0.05. Reciprocal effects between *Ank3* and *Nrxn1* were assessed descriptively from the corresponding virtual-knockout outputs. This analysis was used to generate hypotheses about candidate-associated regulatory programs and was not interpreted as evidence of causal gene regulation *in vivo*.

Upstream transcription-factor perturbation was conducted separately in the embryonic cortical GSE274763 reference dataset using CellOracle v0.20.0 (Kamimoto et al., 2023) and a mouse promoter-motif-constrained gene regulatory network. The analysis was restricted to SOX5+/AUTS2+ immature projection neurons. Motif-supported transcription factors with detectable expression were selected before simulation. For each selected transcription factor, knockout was simulated by fixing its expression at zero, whereas bounded overexpression was simulated by fixing its expression at the 90th percentile of imputed expression. Perturbation effects were propagated for three steps, with *Ank3* and *Nrxn1* treated as downstream readouts. The resulting shifts were summarized across the full subset and the four source samples. Because this embryonic reference dataset was not exposed to clozapine, these simulations were interpreted as model-dependent regulatory hypotheses rather than evidence of clozapine-induced regulation.

### 2.11 Statistical analysis

Post-treatment locomotor distance and novel-object discrimination ratio were analyzed using two-way analysis of variance (ANOVA), with housing condition (SOC or SIR) and drug treatment (vehicle or clozapine) as between-subject factors. Prepulse inhibition (PPI) was analyzed separately at each prepulse intensity (69, 78 and 88 dB) using the same two-way ANOVA framework. The 69-, 78-, and 88-dB analyses were prespecified as intensity-specific endpoints and were interpreted separately. For every endpoint, the main effects of housing and drug and their interaction were reported. All pairwise group comparisons were assessed using Tukey’s honestly significant difference procedure. Data are presented as individual observations with mean ± standard error of the mean (SEM). All tests were two-sided. Statistical analyses were performed in Python 3.10.20 using statsmodels 0.14.6, scipy 1.11.4 and pandas 1.5.3. The raw values exported from the GraphPad Prism file were retained as the analysis record; figures were generated in R 4.6.1 using ggplot2 4.0.3.

## 3 Results

### 3.1 Results of behavioral evaluation

#### 3.1.1 Open-field test

Locomotor distance showed a significant main effect of drug treatment (F (1,34) = 31.166, P < 0.001) and a significant housing-by-drug interaction (F (1,34) = 9.898, P = 0.003), whereas the housing main effect did not reach statistical significance (F (1,34) = 3.864, P = 0.058; Figure 2B). Tukey-adjusted comparisons showed that SIR-vehicle mice travelled farther than SOC-vehicle mice (P = 0.005) and that clozapine reduced locomotor distance in SIR mice (SIR-vehicle vs. SIR-clozapine, P < 0.001). SIR-vehicle mice also differed from SOC-clozapine mice (P < 0.001). Thus, acute clozapine was associated with a marked reduction in locomotor distance in SIR mice. Because a significant drug main effect was also present, this result may reflect both a housing-dependent effect and general drug-related locomotor suppression and should not be interpreted as specific normalization.

#### 3.1.2 Novel object recognition

The discrimination ratio showed a significant main effect of housing (F (1,34) = 16.687, P < 0.001), but neither the drug main effect (F (1,34) = 3.576, P = 0.067) nor the housing-by-drug interaction (F (1,34) = 0.314, P = 0.579) was significant (Figure 2C). Tukey-adjusted comparisons showed lower discrimination ratios in SIR-vehicle mice than in SOC-vehicle mice (P = 0.001) and lower ratios in SIR-clozapine mice than in SOC-clozapine mice (P = 0.012). The within-SIR vehicle-versus-clozapine comparison was not significant (P = 0.345). These results indicate a housing-associated recognition-memory deficit without evidence of acute clozapine rescue under this testing schedule.

#### 3.1.3 Prepulse inhibition

PPI was evaluated separately at each prepulse intensity (Figures 2D–F). At 69 dB, significant main effects of housing (F (1,34) = 10.580, P = 0.003) and drug (F (1,34) = 4.861, P = 0.034) were observed, whereas the housing-by-drug interaction was not significant (F (1,34) = 2.715, P = 0.109; Figure 2D). Tukey-adjusted comparisons indicated lower PPI in SIR-vehicle mice than in SOC-vehicle (P = 0.008) and SOC-clozapine mice (P = 0.003). At 78 dB, the main effects of housing (F (1,34) = 2.532, P = 0.121) and drug (F (1,34) = 2.927, P = 0.096), as well as the housing-by-drug interaction (F (1,34) = 3.733, P = 0.062), were not significant (Figure 2E). At 88 dB, the housing main effect was significant (F (1,34) = 11.016, P = 0.002), whereas the drug main effect (F (1,34) = 0.114, P = 0.737) and interaction (F (1,34) = 0.447, P = 0.508) were not significant; SIR-vehicle mice showed lower PPI than SOC-vehicle mice in the Tukey-adjusted comparison (P = 0.038; Figure 2F). Taken together, the PPI findings indicate housing-associated deficits at selected intensities, but do not support a consistent acute clozapine effect on PPI.

#### 3.1.4 Summary of behavioral findings

Across behavioral domains, acute clozapine had its clearest effect on isolation-associated hyperlocomotion. Recognition memory was not improved, and PPI findings varied by prepulse intensity without a significant housing-by-drug interaction at any individual intensity. The behavioral data therefore support a selective acute behavioral effect rather than generalized reversal of social-isolation-associated abnormalities.

### 3.2 Differential gene expression analysis

Across the 11 PFC libraries, 19.1–22.7 million read pairs were processed per sample and 77.9%–90.4% pseudoaligned to the *Mus musculus* GRCm39, Ensembl release 113 transcriptome. Principal-component analysis explained 29.2% and 22.5% of variance on PC1 and PC2, respectively (Supplementary Figure S1). The two SIR-clozapine samples clustered with the SOC-clozapine libraries, whereas SIR2PFC was separated on PC2 and SOC3PFC did not cluster with the other SOC-vehicle libraries. All libraries were retained in the primary analysis.

At FDR <0.05, the SIR-versus-SOC contrast under vehicle contained 124 genes (16 upregulated and 108 downregulated), the clozapine-versus-vehicle contrast within SOC contained 245 genes (50 upregulated and 195 downregulated), and the clozapine-versus-vehicle contrast within SIR contained 18 genes (9 upregulated and 9 downregulated). Fifty-six genes showed a significant housing-by-drug interaction, including 40 positive and 16 negative interaction coefficients (Figures 3A,B). Fourteen genes differed between SIR and SOC under clozapine.

**FIGURE 3:**
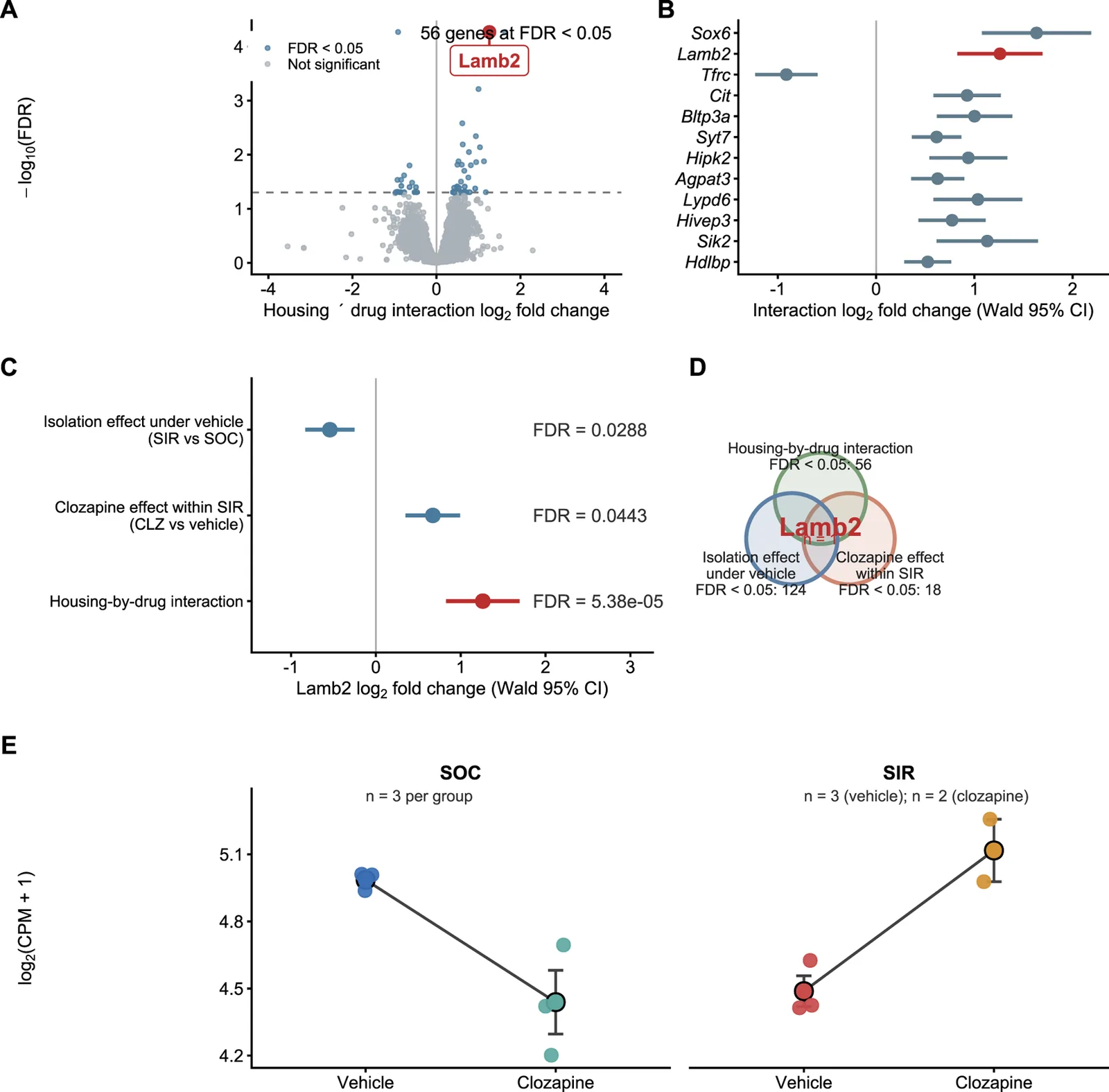
Prioritization of *Lamb2* in the reanalyzed PFC RNA-seq dataset. **(A)** DESeq2 housing-by-drug interaction volcano plot, with *Lamb2* highlighted. **(B)** Effect estimates for the top 12 interaction genes. **(C)** *Lamb2* effect estimates across the isolation, clozapine-within-SIR, and interaction contrasts. **(D)** *Lamb2* was the only gene shared across the three FDR-significant contrast lists. **(E)** Library-size-normalized *Lamb2* expression across the four groups. Each point represents one library; circles and error bars indicate mean and SEM.

*Lamb2* showed a positive housing-by-drug interaction (log2 fold change = 1.261, FDR = 5.38 × 10^-5). After matching FDR-significant results by Ensembl gene ID, *Lamb2* was the only gene shared across the three prespecified DESeq2 contrasts: the isolation effect under vehicle, the clozapine effect within the SIR group, and the housing-by-drug interaction (Figures 3C,D). Library-level *Lamb2* expression across the four groups is shown in Figure 3E. However, the two-contrast directional component of this prioritization was attenuated after omission of SIR2PFC, indicating sensitivity to the small and unbalanced group structure.

*Nrxn1* decreased in SIR mice relative to SOC mice under vehicle conditions and increased after clozapine treatment within SIR mice, but did not reach FDR significance in the factorial model (interaction log2 fold change = 0.268, FDR = 0.485). *Ank3* showed no significant interaction effect (interaction log2 fold change = 0.054, FDR = 0.931). In sensitivity analyses using edgeR quasi-likelihood and limma-voom, no housing-by-drug interaction gene passed FDR correction.

To assess analytic sensitivity, the same four-group factorial design was fitted using edgeR quasi-likelihood and limma-voom. Neither alternative framework identified interaction genes at FDR <0.05. Comparison of interaction coefficients across methods is shown in Supplementary Figures S2A,B, and method-specific results for the primary contrasts and candidate genes are provided in Supplementary Table S1. These analyses show that the FDR-significant interaction list was sensitive to the analytical framework in this small, unbalanced dataset. Enrichment analysis of the 56 FDR-significant housing-by-drug interaction genes did not identify any GO or KEGG term after multiple-testing correction; the lowest observed FDR was 0.736 (Supplementary Table S2). Therefore, these genes did not support a coherent pathway-level interpretation in the primary factorial analysis.

### 3.3 Exploratory enrichment analysis of the historical 286-gene overlap

The historical pairwise DEG contrasts are shown in Figures 4A,B, and their 286-gene overlap and heatmap are shown in Figures 4C,D. GO enrichment analysis of 286 overlapping DEGs revealed significant enrichment in biological processes related to NMDA receptor clustering, axon extension regulation, synaptic vesicle clustering, and synaptic transmission (Figure 4E). Cellular component terms included cell-adhesion complexes, presynaptic cytoskeleton, and postsynaptic density (Figure 4F). Molecular functions involved semaphorin receptor activity and transcription coregulator binding (Figure 4G). KEGG analysis highlighted enrichment in ribosome, cholinergic synapse, and GABAergic synapse pathways (Figure 4H).

**FIGURE 4:**
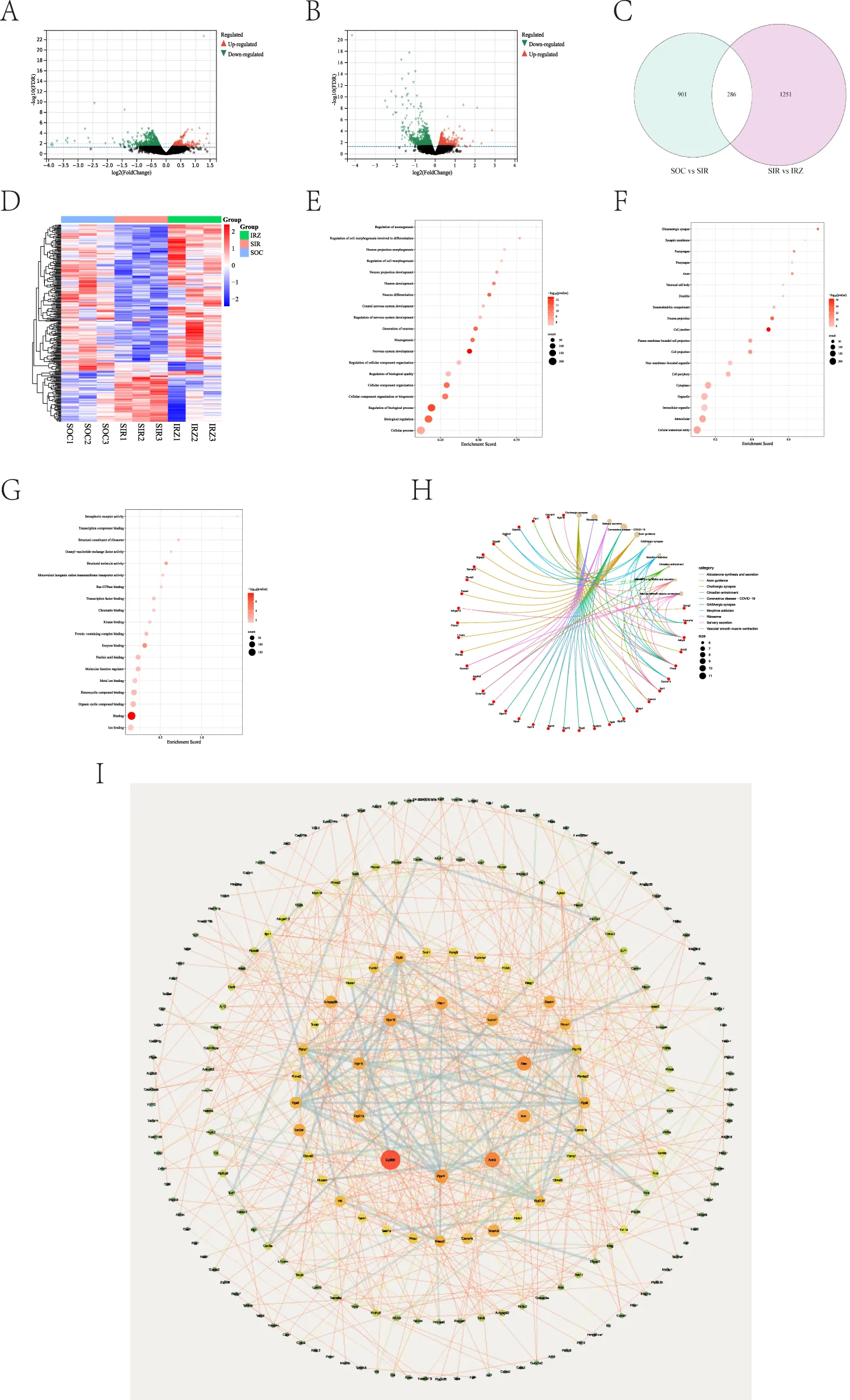
Preliminary analysis of transcriptome sequencing data **(A)** Volcano plot of differentially expressed genes between SOC and SIR groups. **(B)** Volcano plot of differentially expressed genes between SIR-vehicle and SIR-clozapine groups. **(C)** Overlap of the two DEG sets. **(D)** Heatmap of shared candidate genes. **(E–G)** Gene Ontology enrichment analysis for biological process, cellular component, and molecular function. **(H)** KEGG enrichment analysis. **(I)** Protein-protein interaction analysis.

### 3.4 Protein-protein interaction network of DEGs

The protein-protein interaction network included 286 genes from the historical exploratory overlap. These included myelin-associated genes (*Cspg4*, *Plpl*, *Ndrg1*), synapse-related genes (*Arhgap20*, *Nrep*, *Kcnh5*, *Bsn*, *Pclo*), and schizophrenia-relevant genes (*Rasgrf2*, *Hivep1*, *Herc2*, *Wdfy3*) (Figure 4I).

### 3.5 Exploratory computational prioritization of *Ank3* and *Nrxn1*

Random forest, SVM-RFE, LASSO and ElasticNet were applied to the locally retained SIR-vehicle versus SIR-clozapine processed expression matrix. Their intersection prioritized *Ank3* and *Nrxn1*. Because feature selection and performance visualization used the same six samples after prior gene filtering, the analysis cannot provide an unbiased estimate of classification or treatment-response prediction. It is retained solely as an exploratory feature-prioritization procedure. The corresponding feature-selection displays and four-method intersection are shown in Figures 5A–H.

**FIGURE 5:**
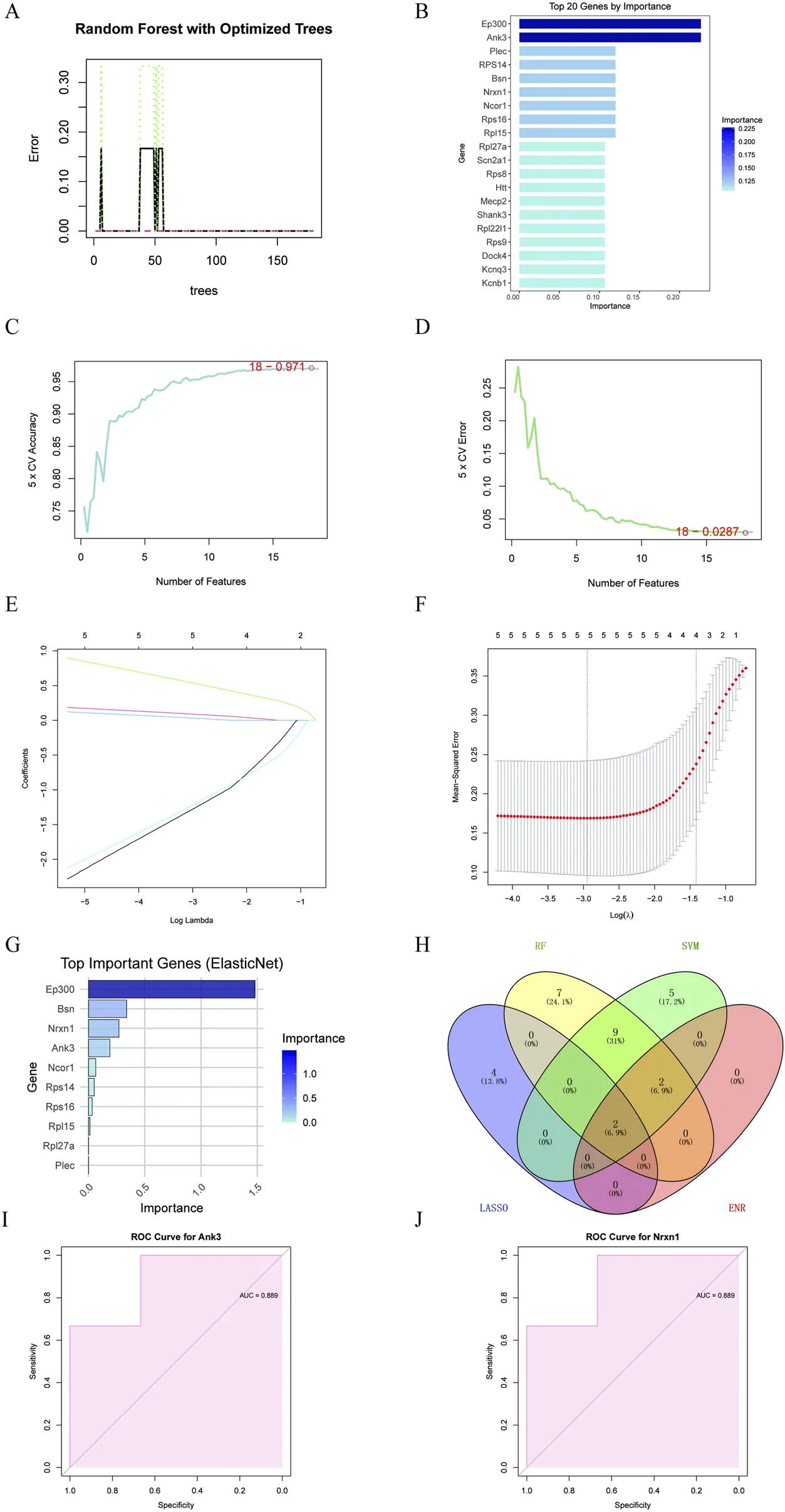
Machine-learning-based candidate gene prioritization and ROC analysis **(A)** Random Forest optimal tree number. **(B)** Top 20 genes ranked by Random Forest importance. **(C)** Accuracy plot of the SVM-RFE algorithm. **(D)** Error-rate plot of the SVM-RFE algorithm. **(E)** LASSO coefficient path. **(F)** Cross-validation curve for LASSO regression. **(G)** Top 10 genes ranked by ElasticNet importance. **(H)** Venn diagram showing the intersection of four machine-learning methods. **(I)** ROC curve for *Ank3*. **(J)** ROC curve for *Nrxn1*.

In the independently reprocessed public libraries, *Nrxn1* decreased in SIR versus SOC under vehicle and increased directionally after clozapine within SIR, but neither contrast survived FDR correction. *Ank3* showed no significant housing, drug or interaction effect. Thus, the factorial RNA-seq model did not validate either candidate.

### 3.6 Descriptive within-dataset candidate visualization

The gene-level ROC curves are shown in Figures 5I,J, whereas the descriptive nomogram displays are provided in Supplementary Figures S3A–C. They describe separation within that matrix but do not estimate external prediction, calibration or clinical utility and were excluded from the primary evidentiary chain.

### 3.7 Exploratory immune-signature scoring

A mouse-specific immune marker matrix was used for exploratory signature scoring in the locally retained expression data. Given the small group size and the use of cortical bulk tissue, these scores were not interpreted as measured immune-cell fractions. No robust association between *Ank3* or *Nrxn1* and the estimated signatures was identified; this analysis was therefore not used to support the proposed mechanism. The exploratory immune-signature plots are shown in Supplementary Figures S4A–D.

### 3.8 Molecular docking analysis of candidate clozapine-responsive proteins

Molecular docking was performed between clozapine and structural models representing *Ank3*-and *Nrxn1*-associated protein regions. The ranked docking energies are summarized in Table 1. The reported values are ranked docking poses from computational searches, not independent biological replicates. These simulations assess geometric and energetic plausibility within the selected structural models and do not establish direct clozapine binding *in vivo*. The docked conformations and two-dimensional interaction diagrams are shown in Figures 6A–D.

**TABLE 1:** Molecular docking results.

Frequency	Protein	Drug	Score (kcal/mol)
1	*Ank3*	Clozapine	−9.4
2	*Ank3*	Clozapine	−7.9
3	*Ank3*	Clozapine	−7.7
4	*Ank3*	Clozapine	−7.7
5	*Ank3*	Clozapine	−7.4
6	*Ank3*	Clozapine	−7.4
7	*Ank3*	Clozapine	−7.3
8	*Ank3*	Clozapine	−7.2
9	*Ank3*	Clozapine	−7.1
1	*Nrxn1*	Clozapine	−7
2	*Nrxn1*	Clozapine	−6.9
3	*Nrxn1*	Clozapine	−6.9
4	*Nrxn1*	Clozapine	−6.9
5	*Nrxn1*	Clozapine	−6.9
6	*Nrxn1*	Clozapine	−6.9
7	*Nrxn1*	Clozapine	−6.9
8	*Nrxn1*	Clozapine	−6.9
9	*Nrxn1*	Clozapine	−6.9

**FIGURE 6:**
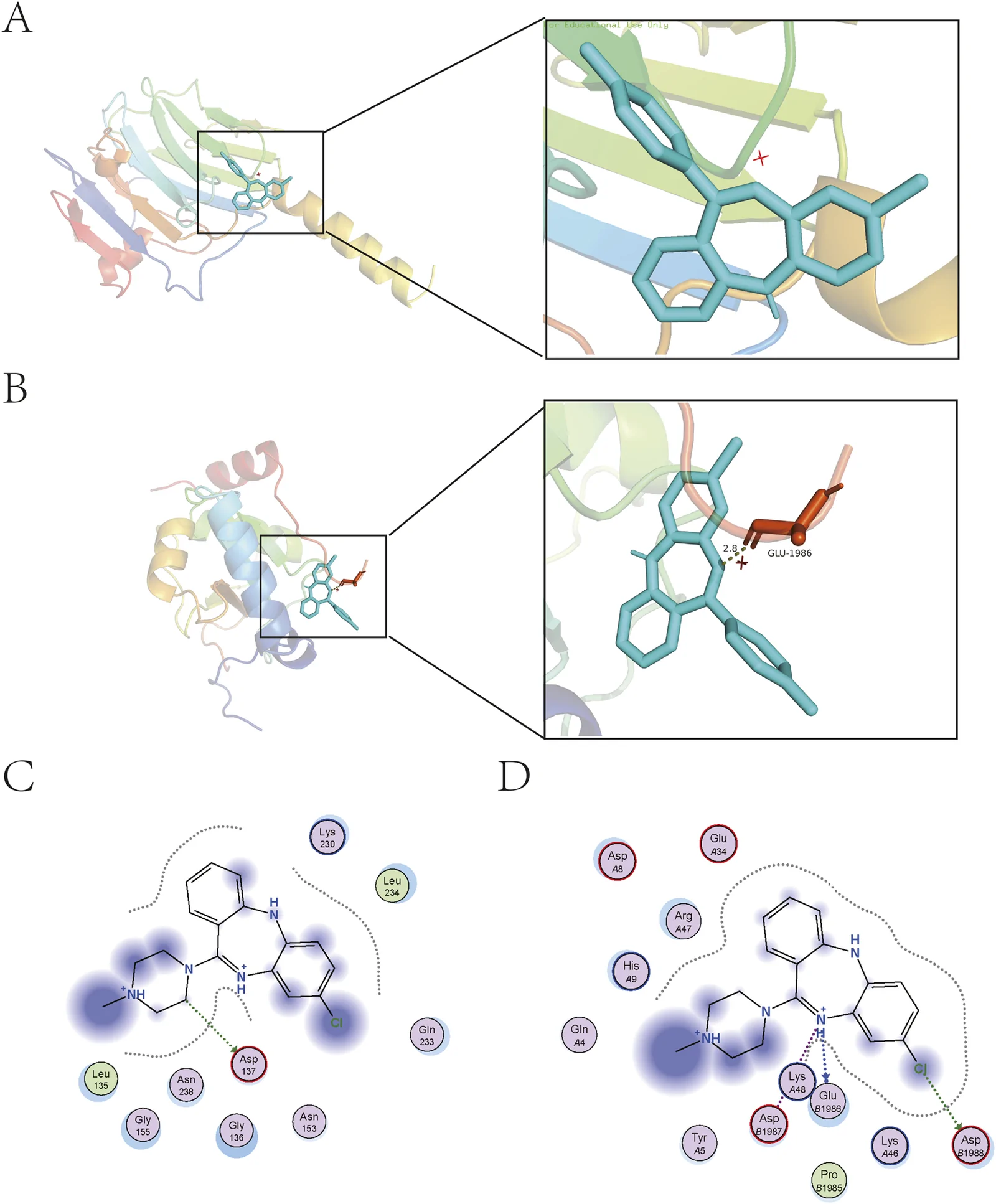
Molecular docking of candidate proteins with clozapine **(A)** Predicted three-dimensional docking interaction between *Nrxn1* and clozapine. **(B)** Predicted three-dimensional docking interaction between *Ank3* and clozapine. **(C)** Two-dimensional interaction diagram for *Nrxn1* and clozapine. **(D)** Two-dimensional interaction diagram for *Ank3* and clozapine.

### 3.9 Molecular dynamics simulation for evaluating the results of molecular docking

#### 3.9.1 Hydrogen bond analysis

Hydrogen-bond analysis was performed to describe potential ligand-protein interactions in the simulated complexes. The number of hydrogen bonds between clozapine and the candidate proteins fluctuated within a limited range of 0–1 during the simulation, suggesting that hydrogen bonding contributed modestly to the predicted binding modes (Figure 7A).

**FIGURE 7:**
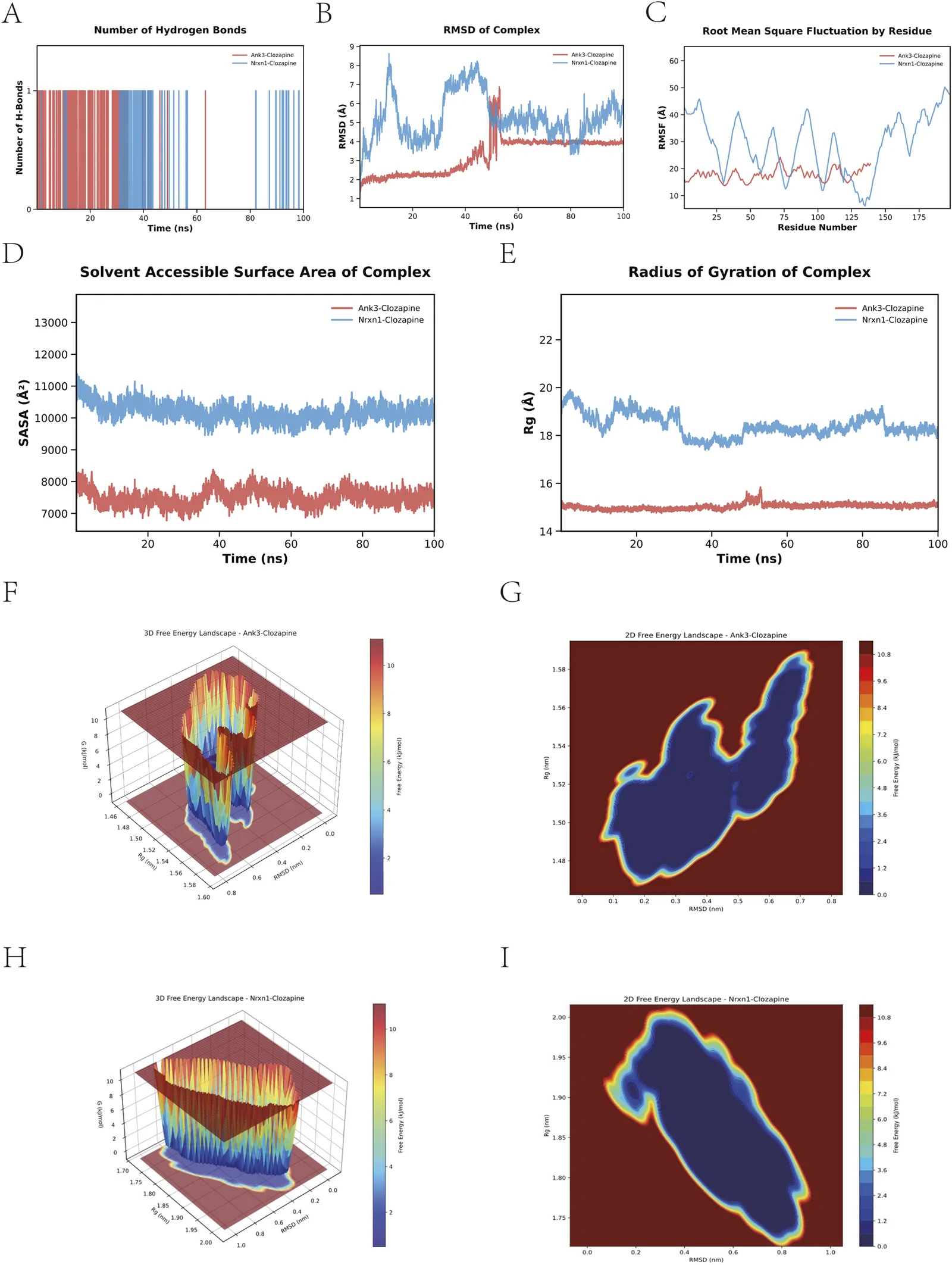
Molecular dynamics simulation **(A–E)** Hydrogen-bond count, root-mean-square deviation, root-mean-square fluctuation, solvent-accessible surface area and radius-of-gyration profiles for the predicted *Ank3*-clozapine and *Nrxn1*-clozapine complexes. **(F,G)** Three- and two-dimensional free-energy landscapes for *Ank3*-clozapine. **(H,I)** Corresponding free-energy landscapes for *Nrxn1*-clozapine.

#### 3.9.2 RMSD analysis

RMSD was used to evaluate the overall stability of the predicted protein-ligand complexes. The *Ank3*-clozapine complex remained largely within approximately 4 Å, whereas the *Nrxn1*-clozapine complex fluctuated around 6 Å (Figure 7B). These trajectories describe the selected structural models and do not establish direct pharmacological targeting.

#### 3.9.3 RMSF analysis

RMSF analysis was used to describe residue-level flexibility. The predicted *Ank3*-clozapine complex showed lower residue fluctuation than the *Nrxn1*-clozapine complex, suggesting lower local mobility in the modeled *Ank3* complex (Figure 7C).

#### 3.9.4 SASA analysis

Solvent-accessible surface area (SASA) was used to track solvent exposure in the modeled complexes. Both trajectories showed transient increases around 40 and 50 ns, indicating sampled conformations with greater solvent-accessible surface area (Figure 7D). These descriptive excursions were interpreted together with RMSD, RMSF, radius of gyration and the free-energy landscapes.

#### 3.9.5 Rg analysis

Radius-of-gyration analysis was used to describe complex compactness. The modeled *Ank3*-clozapine complex remained comparatively stable, whereas the *Nrxn1*-clozapine complex varied more over time (Figure 7E).

#### 3.9.6 MM/GBSA analysis

MM/GBSA estimated binding free energy from representative frames sampled from the final 1 ns of each 100 ns trajectory. The estimates are reported as computational descriptors of the modeled complexes (Table 2).

**TABLE 2:** MM/GBSA analysis of predicted protein-ligand complexes.

Energy type	*Ank3*-clozapine	*Nrxn1*-clozapine
E_VDW	−93.3162 ± 8.0496	−77.0542 ± 6.9459
E_ELE	−15.9687 ± 3.6104	−18.4941 ± 6.3605
E_GB	49.5591 ± 5.0045	37.1899 ± 6.1697
E_SA	−10.5659 ± 0.6389	−8.6337 ± 0.6452
ΔG_binding	−70.2912 ± 8.5358	−66.9921 ± 6.4952

#### 3.9.7 Analysis of free energy landscape

Free-energy landscapes based on RMSD and radius of gyration described the dominant conformational regions of the modeled complexes. Low-energy basins represent more frequently sampled modeled states rather than experimental evidence of protein binding (Figures 7F–I).

### 3.10 Adult PFC cellular context and cell-cell communication analysis

Adult wild-type PFC single-cell RNA-seq data from GSE181021 comprised 5,303 annotated cells from two samples and included excitatory neurons, inhibitory neurons, endothelial cells, astrocytes, microglia, oligodendrocyte-lineage cells and oligodendrocyte precursor cells (Figure 8A). *Lamb2* expression was concentrated in endothelial cells, where it was detected in 263 of 884 cells (29.8%; Figures 8B,E). In contrast, *Ank3* and *Nrxn1* were broadly detected in neuronal populations, including excitatory and inhibitory cells (Figures 8C–E).

**FIGURE 8:**
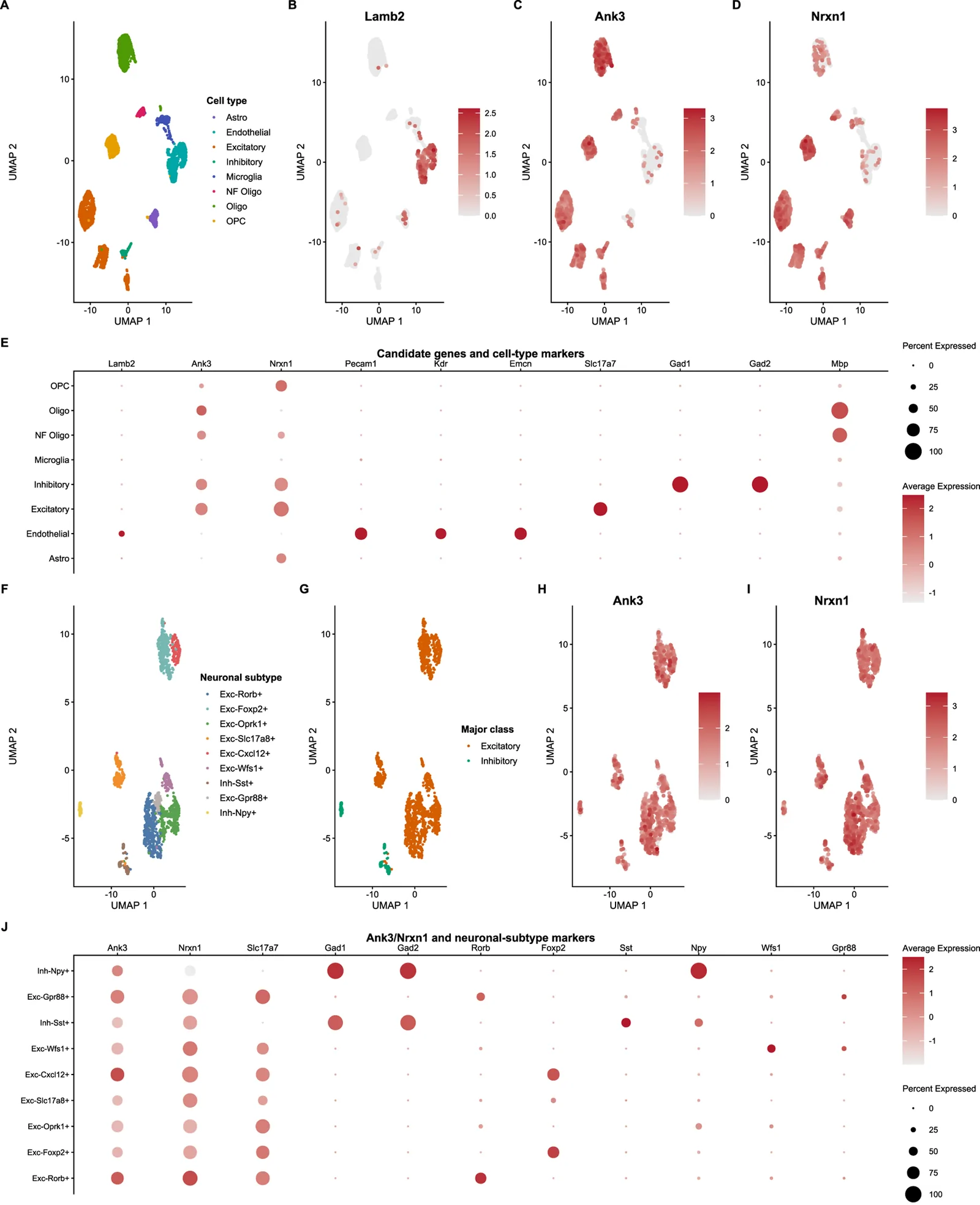
Adult prefrontal-cortex cellular context of *Lamb2*, *Ank3* and *Nrxn1*. **(A)** Major cell-type UMAP. **(B–D)** *Lamb2*, *Ank3* and *Nrxn1* expression. **(E)** Candidate-gene and cell-type marker dot plot. **(F)** Neuronal-subtype UMAP. **(G)** Excitatory and inhibitory neuronal classes. **(H,I)** *Ank3* and *Nrxn1* expression across neuronal populations. **(J)** Candidate-gene and neuronal-subtype marker dot plot. The dataset was not exposed to clozapine and was used for cellular localization.

The neuronal subset contained 1,747 cells and resolved into nine marker-supported excitatory and inhibitory subtypes (Figures 8F,G). *Ank3* and *Nrxn1* were detected across several neuronal subtypes, with prominent expression in excitatory populations (Figures 8H–J).

CellChat analysis of the adult PFC reference identified endothelial-cell-centered LAMININ signaling among major cell classes (Figure 9A). Within the neuronal subset, inferred *Nrxn1*-associated contact communication involved multiple excitatory and inhibitory subtypes (Figure 9B), including predicted interactions received by the Exc-Cxcl12+ subtype (Figure 9C). Because this external dataset was not exposed to clozapine, these analyses provide cellular-context hypotheses and do not demonstrate treatment-induced communication changes.

**FIGURE 9:**
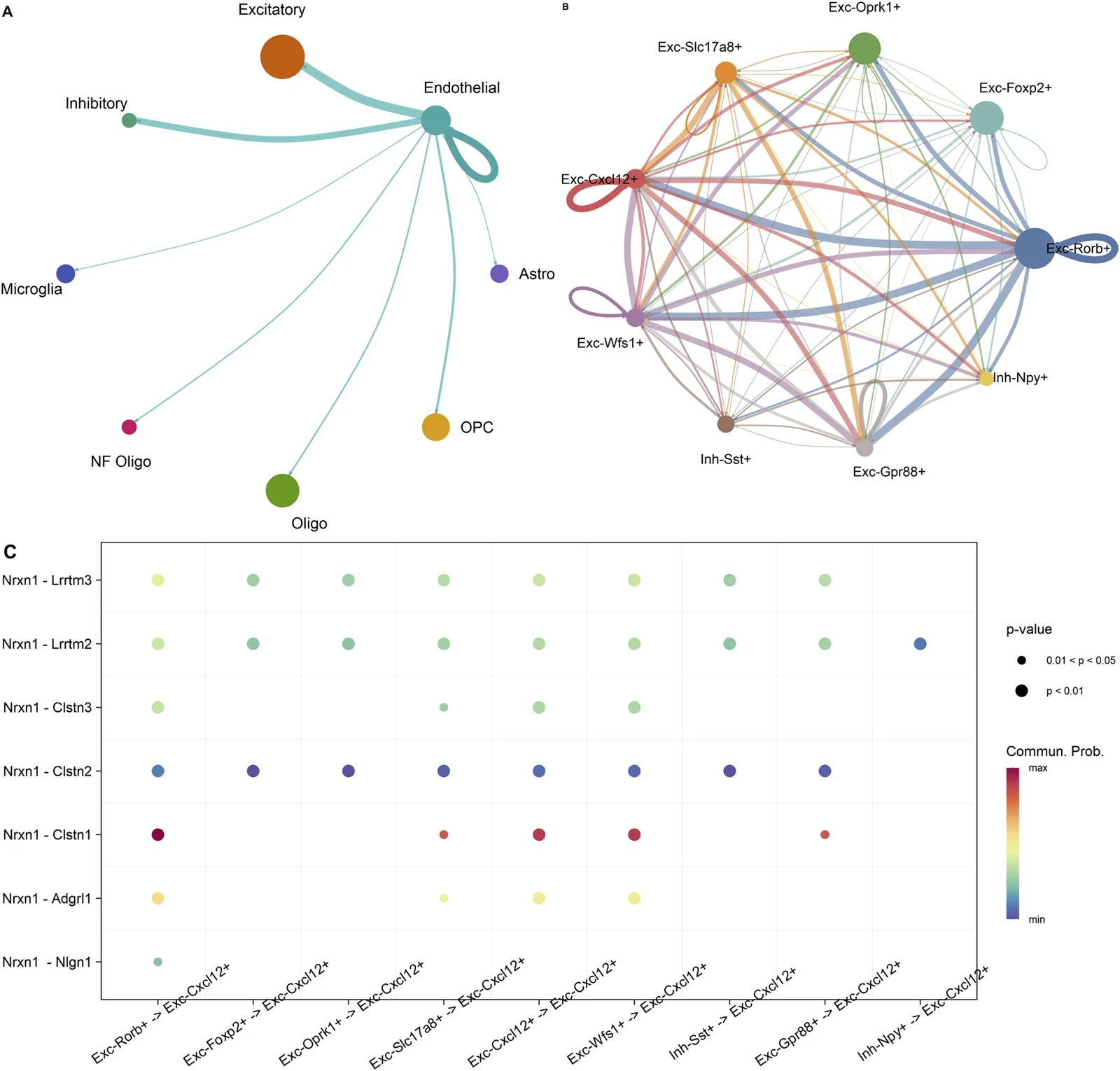
Adult mouse prefrontal cortex cell-cell communication context of *Lamb2* and *Nrxn1*. **(A)** CellChat-inferred *Lamb2*-associated LAMININ signaling among major cell classes, showing endothelial-cell-centered interactions. **(B)** CellChat-inferred *Nrxn1*-associated contact communication among neuronal subtypes. **(C)** Predicted *Nrxn1* ligand-receptor interactions received by the Exc-Cxcl12+ neuronal subtype. Analyses were performed in an external adult wild-type mouse prefrontal cortex single-cell RNA-sequencing dataset and provide cellular-context hypotheses rather than evidence of clozapine-induced communication changes.

### 3.11 In silico perturbation of candidate-associated neuronal programs

In the adult PFC neuronal reference, virtual *Ank3* knockout identified 16 FDR-significant non-target genes and virtual *Nrxn1* knockout identified 15; five genes were shared between the two perturbation profiles (Figures 10A–C). Reciprocal candidate effects were not significant in either direction (both FDR = 1.00), providing no support for a directed *Ank3*-*Nrxn1* regulatory relationship in this adult neuronal reference dataset.

**FIGURE 10:**
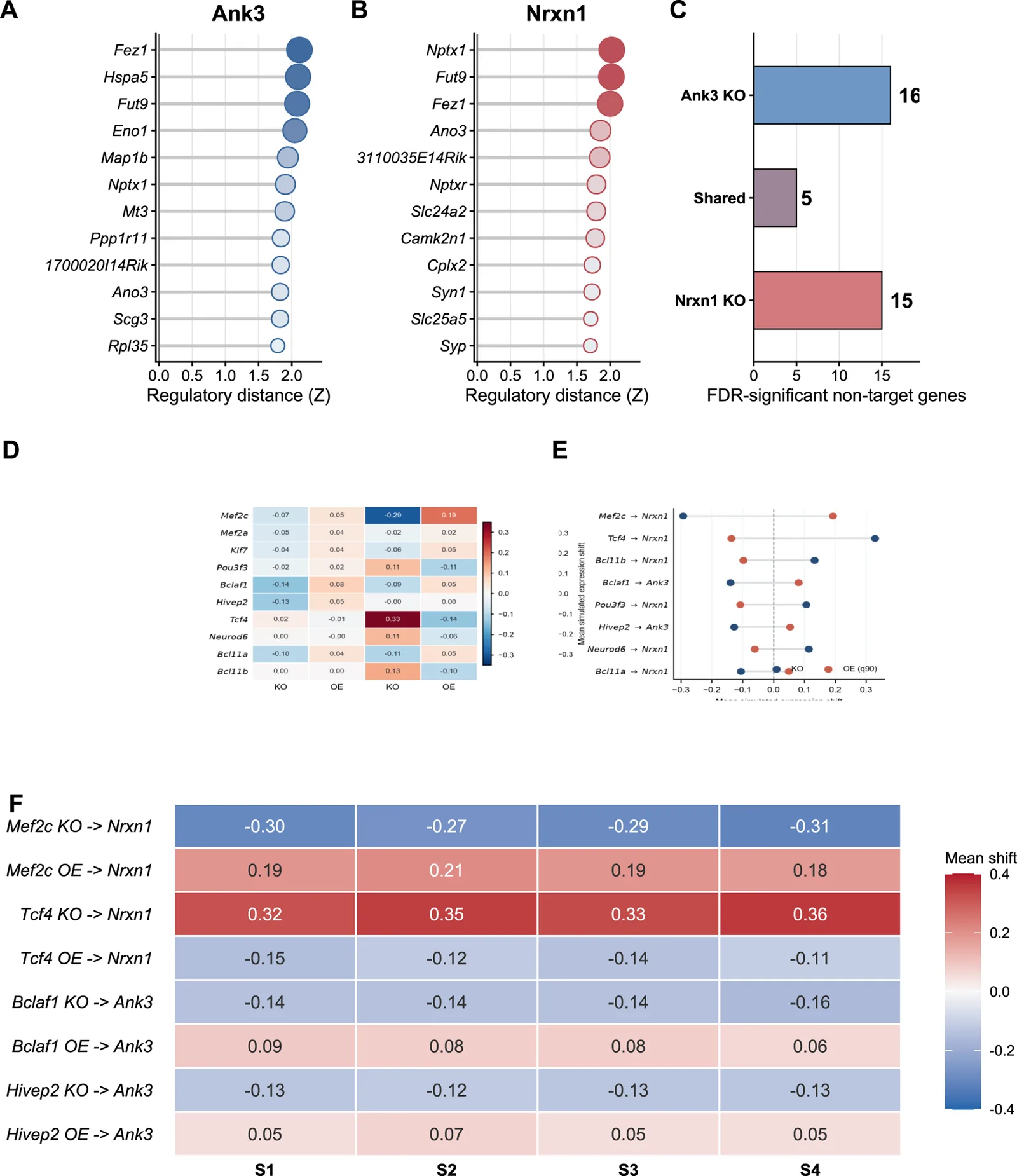
In silico perturbation of *Ank3*-and *Nrxn1*-associated neuronal programs. **(A,B)** Top FDR-significant non-target genes following virtual *Ank3* or *Nrxn1* knockout in adult wild-type mouse prefrontal cortex neurons. **(C)** Numbers of FDR-significant non-target genes identified by each virtual knockout and their overlap. **(D,E)** Simulated expression shifts of *Ank3* and *Nrxn1* following knockout or bounded overexpression of upstream transcription factors in an external embryonic cortical projection-neuron dataset. **(F)** Consistency of the leading transcription-factor perturbation effects across four source samples. These analyses represent external computational hypotheses and do not demonstrate clozapine-induced regulation or direct *Ank3*-*Nrxn1* causality.

CellOracle analysis was performed separately in SOX5+/AUTS2+ immature projection neurons from the embryonic GSE274763 cortical reference. Simulated knockout and bounded overexpression of selected upstream transcription factors produced opposite-direction shifts in the candidate readouts (Figures 10D,E). Mef2c showed the clearest positive-pattern effect on *Nrxn1* (knockout, −0.293; bounded overexpression, +0.193), whereas Tcf4 showed the strongest inverse pattern (knockout, +0.329; bounded overexpression, −0.137). Bclaf1 and Hivep2 showed positive-pattern effects on *Ank3* (Bclaf1, -0.140/+0.081; Hivep2, -0.128/+0.054 for knockout/bounded overexpression). The leading patterns were consistent across the four source samples (Figure 10F). These simulations identify model-dependent transcription-factor sensitivity in an external embryonic neuronal context; they do not establish *in vivo* regulation, direct *Ank3*-*Nrxn1* causality, or clozapine-induced effects.

## 4 Discussion

In this study, a social isolation-rearing model was used to examine schizophrenia-relevant behavioral phenotypes and molecular changes associated with acute clozapine exposure in the prefrontal cortex. Reanalysis of the behavioral data as a four-group factorial experiment resolved inconsistencies in the original statistical reporting and allowed housing, drug, and housing-by-drug interaction effects to be distinguished explicitly.

Acute clozapine produced a domain-selective behavioral profile. The clearest effect was a housing-by-drug interaction in locomotor distance, driven by a marked reduction among SIR mice. A smaller, non-significant reduction in SOC mice indicates that general locomotor suppression may have contributed to this effect. Recognition memory was not improved. PPI showed housing-associated differences at selected prepulse intensities, but no significant housing-by-drug interaction at an individual intensity.

The behavioral profile fits an acute pharmacological challenge with greater sensitivity in locomotor and sensorimotor-gating measures than in recognition memory. Because the design did not separate therapeutic-like activity from nonspecific motor effects, the downstream molecular results are best read in the context of this selective, short-term response rather than generalized behavioral rescue.

Factorial reanalysis of all verifiable public PFC libraries substantially narrowed the transcriptomic claims. Fifty-six genes showed a housing-by-drug interaction at FDR <0.05, and *Lamb2* was the only gene meeting the primary two-contrast directional criterion, although that result was sensitive to omission of the PCA-atypical SIR2PFC sample. Directionally concordant overlap of pairwise DEG lists was therefore an unstable approximation of molecular rescue in these small, unbalanced groups.

The previous network and machine-learning workflow prioritized *Ank3* and *Nrxn1* . *Lamb2* emerged only after the revised factorial reanalysis and was therefore examined separately through adult cellular localization and exploratory endothelial virtual knockout rather than being retroactively incorporated into the historical docking and molecular-dynamics workflow (Supplementary Figure S5). *Nrxn1* remains biologically relevant to neurodevelopmental and synaptic phenotypes, although the present dataset does not establish its clozapine responsiveness (Fernando et al., 2025). In the factorial DESeq2 model, neither gene met the FDR threshold for a housing-by-drug interaction. *Nrxn1* retained a biologically coherent directional pattern across influence analyses, whereas *Ank3* lacked a consistent differential-expression signal. We therefore treat both genes as candidate signals nominated by computational prioritization; *Nrxn1* has the stronger directional support, but neither qualifies as a predictive biomarker on these data.

The ROC and nomogram displays summarize separation within the same small matrix used for feature selection; their estimates describe the discovery set rather than external predictive performance. Docking and molecular-dynamics simulations serve a similarly descriptive role by specifying structural hypotheses for subsequent testing.

Transcriptomic enrichment implicated synaptic organization, neurotransmitter handling and GABAergic-related processes. These associations nominate pathways that may respond to acute clozapine exposure. Direct measures of synaptic function, receptor distribution and circuit plasticity were outside the study design.

Mouse immune-signature scores varied across the small local expression matrix. These values represent relative expression patterns rather than measured immune-cell proportions. Their weak associations with *Ank3* and *Nrxn1* offered no support for an immune mechanism in this study.

Docking and molecular-dynamics simulations described the behavior of clozapine within selected *Ank3* -and *Nrxn1* -associated structural models. The trajectories characterize those models and define testable binding hypotheses; confirmation of physical binding *in vivo* requires direct biochemical evidence. This caution is particularly important because docking scores and modeled poses do not substitute for experimental evidence of molecular recognition (Tosstorff and Kuhn, 2025).

The revised external single-cell analyses were designed to distinguish cellular localization from regulatory simulation. In adult wild-type PFC data, *Lamb2* was concentrated in endothelial cells, whereas *Ank3* and *Nrxn1* were broadly detected across neuronal populations and neuronal subtypes. CellChat provided an adult PFC communication context, with endothelial-cell-centered LAMININ signaling and *Nrxn1*-associated contact communication across neuronal subtypes. These results support cell-type-specific hypotheses for the bulk RNA-seq candidates, but the external reference was not exposed to clozapine and cannot validate a treatment-associated expression change.

Virtual knockout in the adult neuronal reference identified partially overlapping *Ank3* and *Nrxn1* perturbation profiles, but neither candidate showed a significant reciprocal perturbation effect. This result does not support a directed *Ank3*-*Nrxn1* regulatory relationship in the inferred adult neuronal network. CellOracle analysis was conducted separately in an embryonic projection-neuron reference and identified model-dependent sensitivity of *Nrxn1* to Mef2c and Tcf4 perturbation and of *Ank3* to Bclaf1 and Hivep2 perturbation. These simulations provide prioritized regulatory hypotheses, while remaining conditional on the inferred network, the external developmental context and the selected simulation parameters.

Taken together, the revised analyses define selective behavioral and prefrontal transcriptomic correlates of an acute clozapine challenge. *Lamb2* was the strongest FDR-supported transcriptomic signal in the primary factorial model. By contrast, *Nrxn1* retained only directionally consistent support, and *Ank3* lacked factorial-model support; both therefore remain exploratory candidates from the historical pairwise and computational workflow. The external adult and embryonic single-cell analyses provide cellular and regulatory hypotheses for future testing rather than evidence of clozapine-responsive causal mechanisms.

Interpretation is constrained by the acute dosing schedule, male-only cohort and restriction to PFC tissue. Acute clozapine exposure can produce early molecular changes in rodent brain tissue (Robbins et al., 2008; Korlatowicz et al., 2021). Because PFC tissue was collected approximately 2.5–3 h after the final administration and after the final behavioral session, the transcriptomic findings represent a post-behavioral acute-exposure snapshot rather than a pharmacokinetic peak or sustained-treatment state. Retrospective qPCR validation was not possible because no suitable archived PFC tissue from the sequenced animals remained after completion of the original study. Molecular groups were small, one locally documented SIR-clozapine library was not publicly available, and the interaction results were sensitive to alternative differential-expression frameworks. The adult and embryonic single-cell datasets were external references without clozapine exposure; their localization, communication and perturbation outputs therefore provide computational context rather than independent validation of clozapine-responsive molecular changes.

Within these constraints, the study links a selective acute behavioral profile to PFC molecular changes in the social-isolation model. The strongest conclusions concern housing-dependent behavior and FDR-supported transcriptomic interactions; *Ank3* and *Nrxn1* remain experimentally testable candidates rather than response biomarkers.

## 5 Conclusion

In socially isolated mice, acute clozapine produced a selective behavioral profile accompanied by PFC transcriptional changes. Factorial reanalysis of publicly retrievable libraries identified FDR-supported housing-by-drug interaction signals, with *Lamb2* as the strongest transcriptomic candidate. *Nrxn1* retained directionally consistent but FDR-nonsignificant support, whereas *Ank3* lacked factorial-model support. Adult PFC single-cell analyses localized *Lamb2* predominantly to endothelial cells and *Ank3*/*Nrxn1* to neuronal populations, while virtual perturbation analyses generated external regulatory hypotheses. These findings require independent molecular and functional validation and do not establish causal mechanisms of clozapine response.

## Data Availability

The publicly available datasets analyzed in this study can be accessed through the NCBI Sequence Read Archive under accession number PRJNA1014397 and the Gene Expression Omnibus under accession numbers GSE181021 and GSE274763. Additional processed data supporting the findings are available from the corresponding author upon reasonable request.
