# Supplementary material for: Selective behavioral and prefrontal transcriptomic correlates of acute clozapine exposure in socially isolated mice
Source: Front Pharmacol. 2026 Sep 28;17:1944363. doi: 10.3389/fphar.2026.1944363 (PMC13655524; doi:10.3389/fphar.2026.1944363)
Supplement: Supplementary file 1 [file Supplementaryfile1.pdf]

Supplementary Figure Legends

Supplementary Fig. S1. Principal-component analysis of publicly retrievable PFC RNA-seq libraries.

Variance-stabilized gene-level counts from the primary DESeq2 workflow were summarized using the 500 most variable genes. PC1 and PC2 explained 29.2% and 22.5% of variance, respectively. All 11 publicly retrievable libraries were retained in the primary factorial analysis. Dashed enclosures show the within-group distribution; SIR2PFC was retained despite its separation from the other SIR-vehicle libraries.

Supplementary Fig. S2. Concordance of housing-by-drug interaction effect estimates across differential-expression methods.

(A) DESeq2 interaction log2 fold changes are compared with edgeR quasi-likelihood estimates. (B) DESeq2 interaction log2 fold changes are compared with limma-voom estimates. Each point represents one gene, and the dashed line indicates identity. Lamb2, Nrxn1 and Ank3 are highlighted. Although effect estimates were directionally concordant for the highlighted genes, no interaction gene passed FDR < 0.05 in the edgeR quasi-likelihood or limma-voom analyses.

Supplementary Fig. S3. Exploratory nomogram analysis.

(A) Nomogram based on the two candidate clozapine-responsive genes. (B) ROC curve of the nomogram. (C) Calibration curve of the nomogram.

Supplementary Fig. S4. Correlation analysis of immune infiltration and immune cells.

(A) Stacked bar chart of estimated immune cell infiltration proportions. (B) Immune cell correlation map. (C) Immune infiltration heatmap. (D) Correlation map of Ank3 and Nrxn1 with estimated immune cell populations.

Supplementary Fig. S5. Lamb2 localization and virtual knockout in adult mouse prefrontal cortex.

(A) Lamb2-positive-cell proportions across major cell classes in two adult wild-type mouse prefrontal cortex samples, showing the highest detection in endothelial cells. Point size represents the number of cells. (B) Virtual Lamb2 knockout using the endothelial-cell input identified Gpm6a as the only FDR-significant non-target gene. The analysis provides an exploratory cellular-context hypothesis rather than evidence of clozapine-induced regulation.

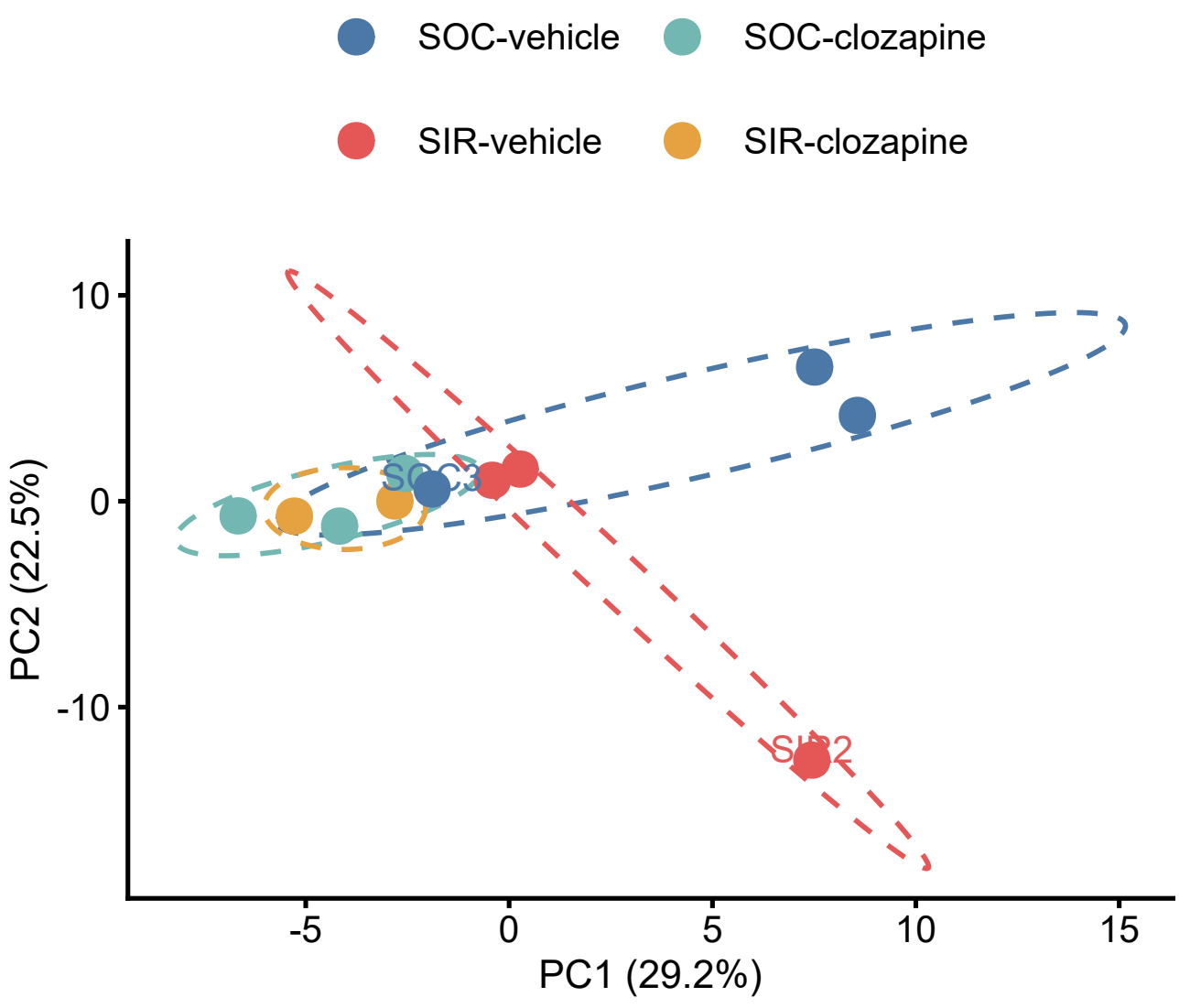

SOC-vehicle
SOC-clozapine
SIR-vehicle
SIR-clozapine
SOC3
SIR2
10
0
-10
-5
0
5
10
15
PC2 (22.5%)
PC1 (29.2%)

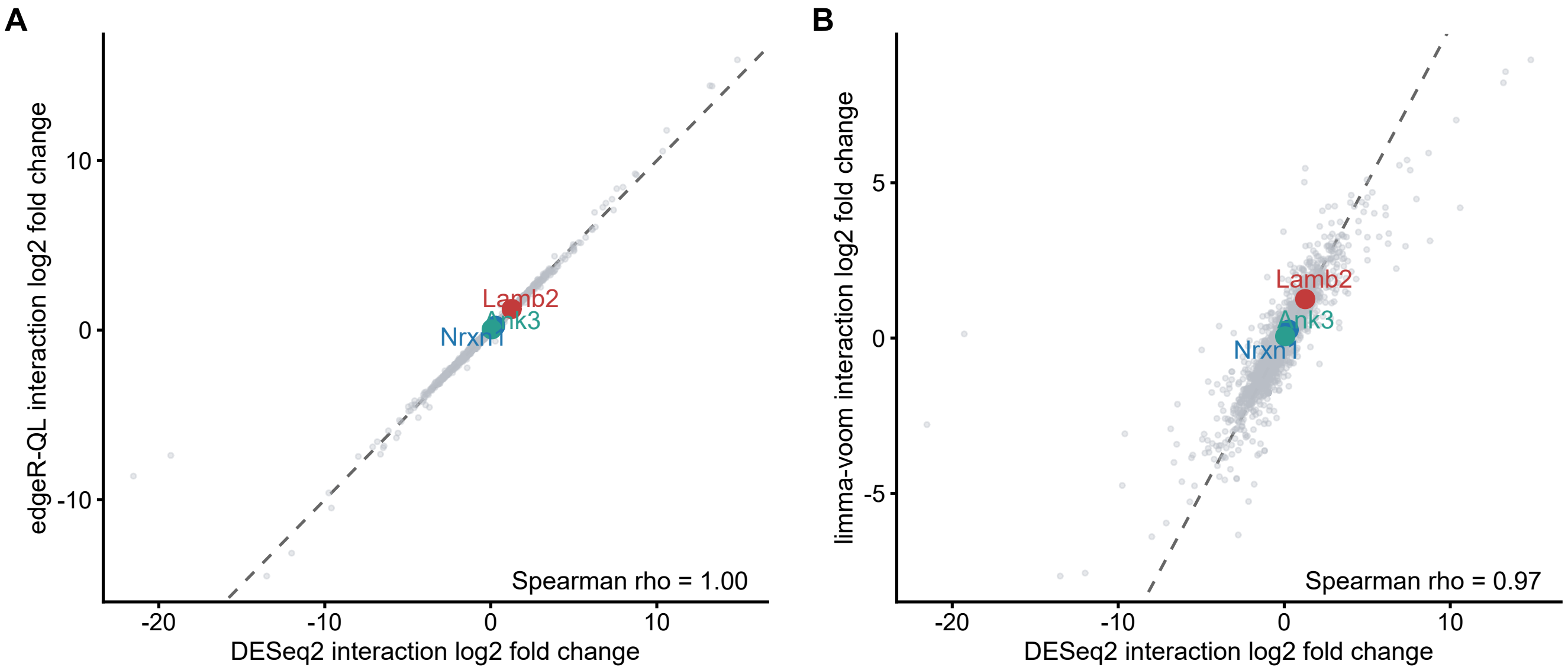
A
edgeR-QL interaction log2 fold change
DESeq2 interaction log2 fold change
10
0
-10
-20
Lamb2
Ank3
Nrxn1
Spearman rho = 1.00
B
limma-voom interaction log2 fold change
DESeq2 interaction log2 fold change
5
-5
Spearman rho = 0.97

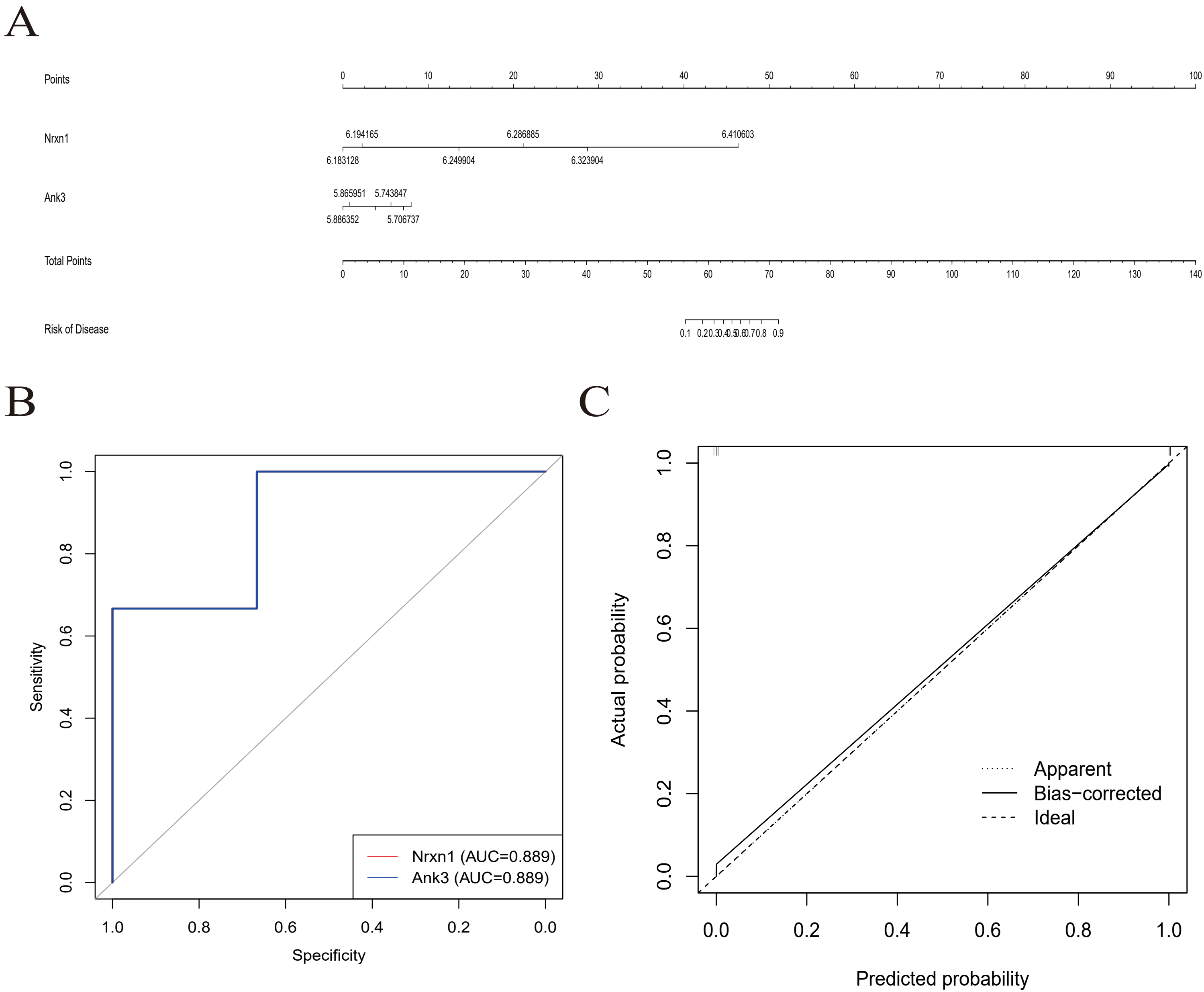
A
Points
0
10
20
30
40
50
60
70
80
90
100
Nrxn1
6.194165
6.286885
6.410603
6.183128
6.249904
6.323904
Ank3
5.865951
5.743847
5.886352
5.706737
Total Points
0
10
20
30
40
50
60
70
80
90
100
110
120
130
140
Risk of Disease
0.1
0.2
0.3
0.4
0.5
0.6
0.7
0.8
0.9
B
Sensitivity
Specificity
1.0
0.8
0.6
0.4
0.2
0.0
Nrxn1 (AUC=0.889)
Ank3 (AUC=0.889)
C
Actual probability
Predicted probability
0.0
0.2
0.4
0.6
0.8
1.0
Apparent
Bias−corrected
Ideal

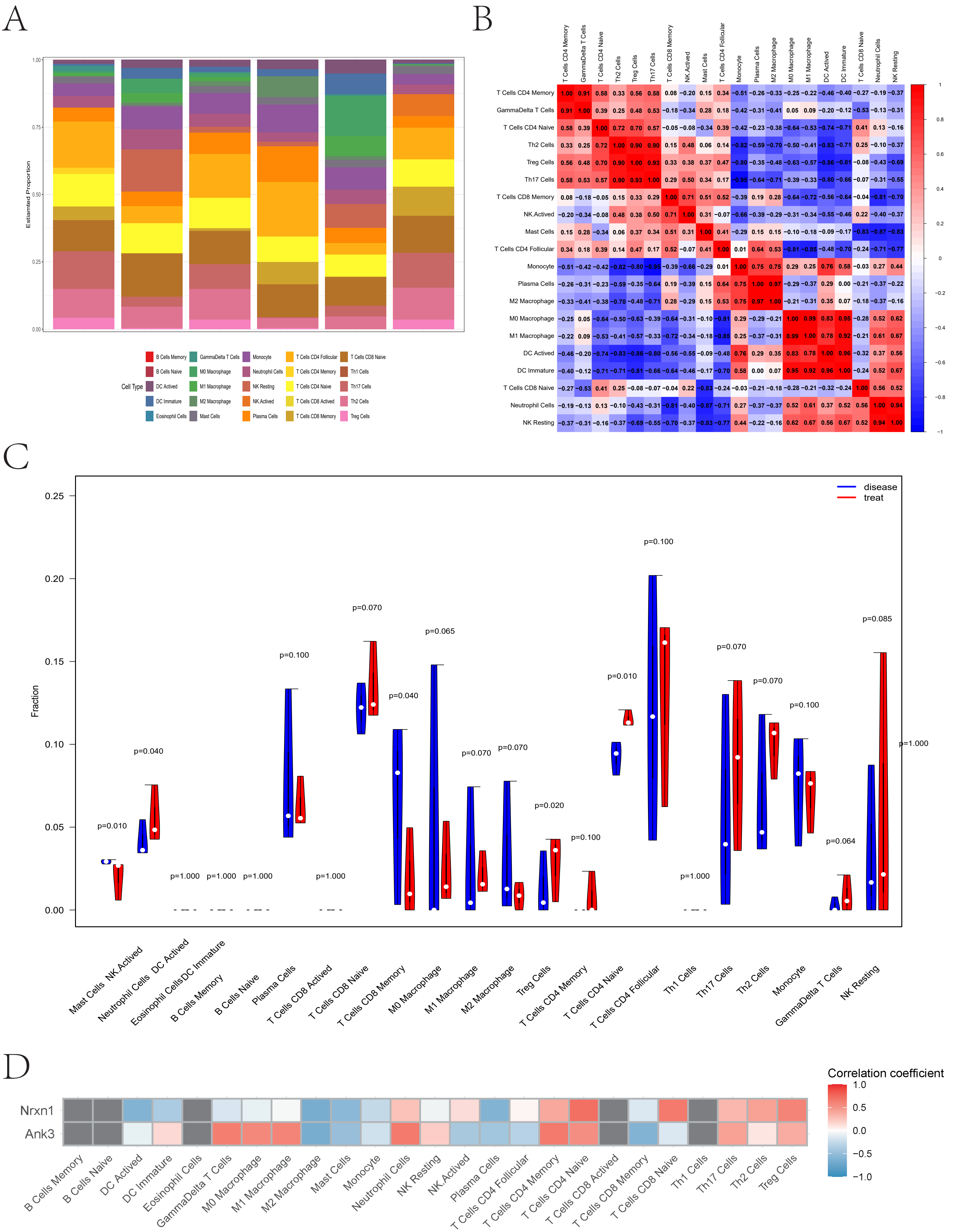
A
B
C
D
Estiamted Proportion
1.00
0.75
0.50
0.25
0.00
Cell Type
B Cells Memory
B Cells Naive
DC Actived
DC Immature
Eosinophil Cells
GammaDelta T Cells
M0 Macrophage
M1 Macrophage
M2 Macrophage
Mast Cells
Monocyte
Neutrophil Cells
NK Resting
NK.Actived
Plasma Cells
T Cells CD4 Follicular
T Cells CD4 Memory
T Cells CD4 Naive
T Cells CD8 Actived
T Cells CD8 Memory
T Cells CD8 Naive
Th1 Cells
Th17 Cells
Th2 Cells
Treg Cells
disease
treat
Fraction
0.25
0.20
0.15
0.10
0.05
0.00
p=0.010
p=0.040
p=1.000
p=1.000
p=1.000
p=0.100
p=1.000
p=0.070
p=0.040
p=0.065
p=0.070
p=0.070
p=0.020
p=0.100
p=0.010
p=0.100
p=1.000
p=0.070
p=0.070
p=0.100
p=0.064
p=0.085
p=1.000
Mast Cells
NK.Actived
Neutrophil Cells
DC Actived
Eosinophil Cells
DC Immature
B Cells Memory
B Cells Naive
Plasma Cells
T Cells CD8 Actived
T Cells CD8 Naive
T Cells CD8 Memory
M0 Macrophage
M1 Macrophage
M2 Macrophage
Treg Cells
T Cells CD4 Memory
T Cells CD4 Naive
T Cells CD4 Follicular
Th1 Cells
Th17 Cells
Th2 Cells
Monocyte
GammaDelta T Cells
NK Resting
Correlation coefficient
1.0
0.5
0.0
−0.5
−1.0
Nrxn1
Ank3

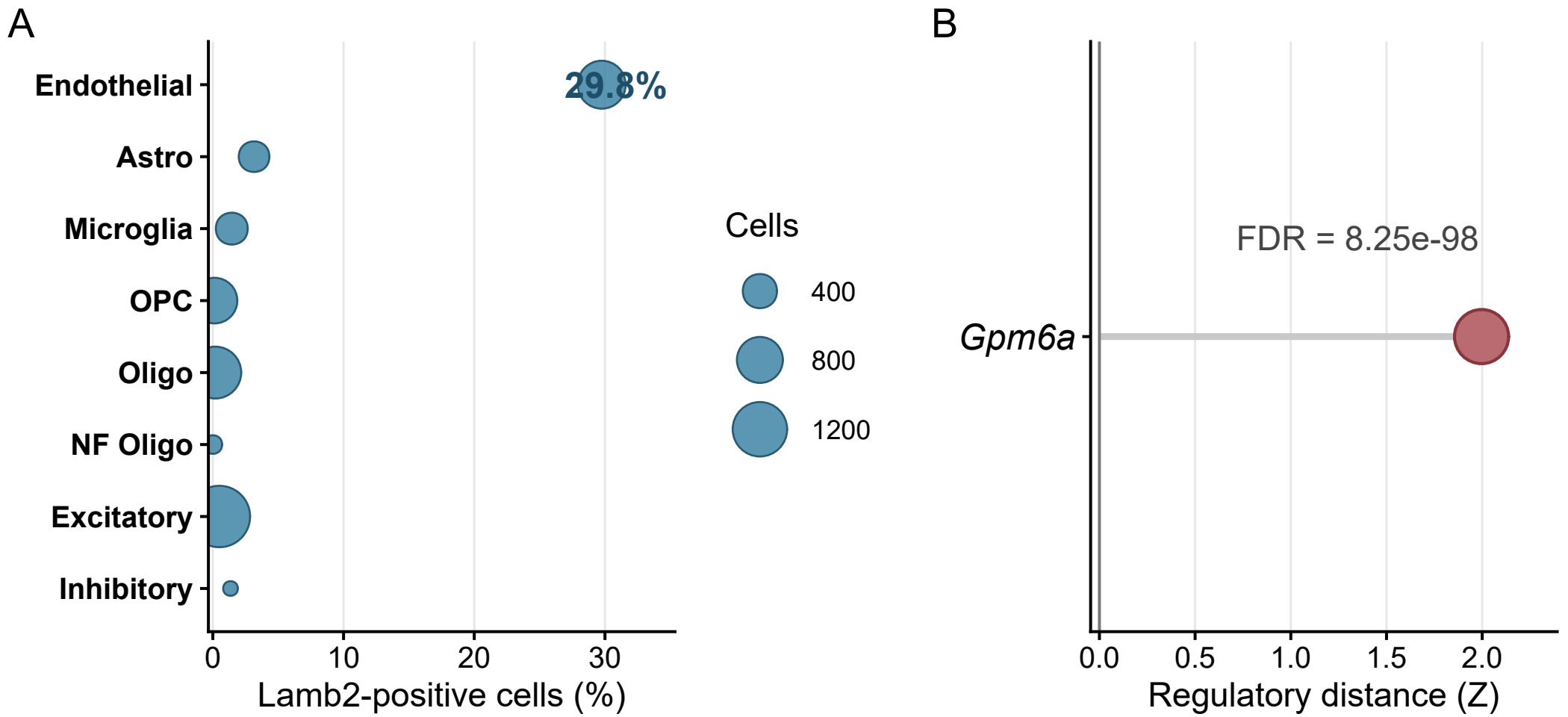

A
Endothelial
Astro
Microglia
OPC
Oligo
NF Oligo
Excitatory
Inhibitory
29.8%
0
10
20
30
Lamb2-positive cells (%)
Cells
400
800
1200
B
FDR = 8.25e-98
Gpm6a
0.0
0.5
1.0
1.5
2.0
Regulatory distance (Z)

Supplementary Table S1

Table S1a. Numbers of genes meeting FDR < 0.05 across differential-expression methods and factorial contrasts.

Method	Contrast	Genes tested	Genes with FDR < 0.05
DESeq2	Interaction	16084	56
DESeq2	Housing under vehicle	16084	124
DESeq2	Drug within soc	16084	245
DESeq2	Drug within sir	16084	18
DESeq2	Housing under clozapine	16084	14
edgeR quasi-likelihood	Interaction	15994	0
edgeR quasi-likelihood	Housing under vehicle	15994	0
edgeR quasi-likelihood	Drug within soc	15994	0
edgeR quasi-likelihood	Drug within sir	15994	0
edgeR quasi-likelihood	Housing under clozapine	15994	0
limma-voom	Interaction	15994	0
limma-voom	Housing under vehicle	15994	0
limma-voom	Drug within soc	15994	0
limma-voom	Drug within sir	15994	0
limma-voom	Housing under clozapine	15994	0

Table S1b. Housing-by-drug interaction estimates for Lamb2, Nrxn1 and Ank3 under three differential-expression methods.

Gene	Method	Interaction log2 fold change	FDR
Lamb2	DESeq2	1.261	5.38e-05
Lamb2	edgeR quasi-likelihood	1.25	2.49e-01
Lamb2	limma-voom	1.254	2.21e-01
Nrxn1	DESeq2	0.268	4.85e-01
Nrxn1	edgeR quasi-likelihood	0.263	6.00e-01
Nrxn1	limma-voom	0.268	6.36e-01
Ank3	DESeq2	0.054	9.31e-01
Ank3	edgeR quasi-likelihood	0.049	9.47e-01
Ank3	limma-voom	0.055	9.46e-01

Note: The same housing-by-drug factorial design was fitted with each method. FDR values were calculated using the Benjamini-Hochberg procedure.

Supplementary Table S2. Summary of enrichment analysis for FDR-significant housing-by-drug interaction genes

The 56 genes with FDR < 0.05 for the housing-by-drug interaction were tested against the 16,084 genes retained in the primary DESeq2 analysis. GO biological process, cellular component, molecular function and KEGG terms were tested separately. P values were adjusted using the Benjamini-Hochberg procedure.

Category	Terms tested	Lowest FDR	FDR-significant terms
GO biological process	6,013	1.000	0
GO cellular component	772	0.915	0
GO molecular function	1,181	0.736	0
KEGG	204	1.000	0

Abbreviations: FDR, false discovery rate; GO, Gene Ontology; KEGG, Kyoto Encyclopedia of Genes and Genomes.

Supplementary Table S3. Software, versions, sources, and analytical use in the revised study.

Analysis stage	Software/package	Version	Primary use	Source/reference
Original RNA-seq read processing	Trimmomatic	0.39	Adapter and quality trimming of sequencing reads.	Bolger et al., 2014
Original RNA-seq alignment	HISAT2	2.1.0	Alignment of cleaned reads to the mouse reference genome.	Kim et al., 2015
Original RNA-seq transcript assembly	StringTie	2.1.4	Transcript assembly and abundance estimation.	Pertea et al., 2015
Public PFC RNA-seq quantification	kallisto	0.50.1	Pseudoalignment and transcript quantification of the 11 publicly retrievable PFC libraries.	Bray et al., 2016; run_info.json
Reference annotation	Ensembl Mus musculus	GRCm39, release 113	Transcript-to-gene mapping for the revised public PFC reanalysis.	Ensembl release 113
Count import	tximport	1.40.0	Gene-level import of kallisto abundance estimates using lengthScaledTPM.	Soneson et al., 2015
Primary differential expression	DESeq2	1.52.0	Four-group housing x drug factorial model and prespecified contrasts.	Love et al., 2014
Sensitivity differential expression	edgeR	4.10.1	Quasi-likelihood sensitivity analysis using the same factorial design.	Robinson et al., 2010
Sensitivity differential expression	limma	3.68.4	voom-based sensitivity analysis using the same factorial design.	Law et al., 2014; Ritchie et al., 2015
Single-cell RNA-seq reanalysis	Seurat	5.5.1	Quality control, normalization, dimensional reduction, clustering, and visualization of public single-cell data.	Hao et al., 2024
Cell-cell communication	CellChat	2.2.0.9001	Inference and visualization of ligand-receptor communication in the adult PFC reference.	Jin et al., 2021
Virtual knockout	scTenifoldKnk	1.0.3	Exploratory gene-regulatory perturbation analysis for Ank3, Nrxn1, and Lamb2 in adult neuronal or endothelial contexts.	Osorio et al., 2022
Transcription-factor perturbation	CellOracle	0.20.0	Motif-constrained exploratory transcription-factor perturbation in the embryonic cortical reference.	Kamimoto et al., 2023
Molecular docking	AutoDockTools	1.5.6	Preparation of protein and ligand structures for exploratory docking.	Morris et al., 2009
Molecular dynamics	Amber	24	Simulation and trajectory analysis of the Ank3-clozapine and Nrxn1-clozapine complexes.	Amber 24 software documentation; archived analysis record

Note: Version numbers for the revised public-data analyses were recovered from run manifests, saved scripts, or package metadata. The original experimental RNA-seq processing records provided by the laboratory were used for the historical workflow entries. All docking, molecular-dynamics, single-cell, and virtual-perturbation analyses are exploratory computational analyses and do not establish direct binding or causal regulation.
